# Supplementary figures and images for: Longitudinally monitored immune biomarkers predict the timing of COVID-19 outcomes
Source: PLoS Comput Biol. 2022 Jan 18;18(1):e1009778. doi: 10.1371/journal.pcbi.1009778 (PMC8812869; doi:10.1371/journal.pcbi.1009778)

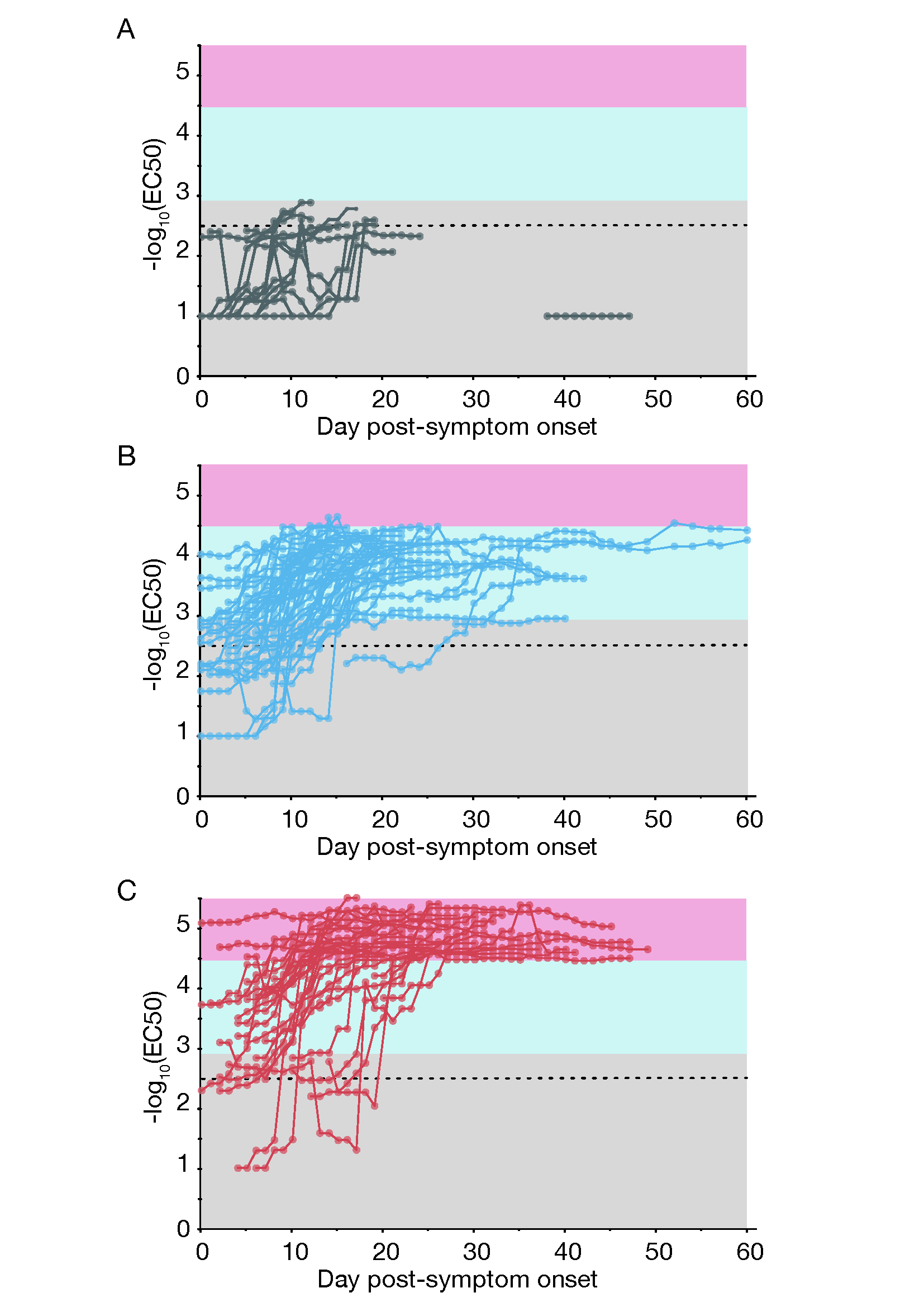

Supplement: S1 Fig — EC50 titers over time were used to categorize patients with ≥ 7 days of hospitalization (n = 130) into three categories that describe the sustained IgG titer: (A) low, (B) medium and (C) high. Categorized patients are required to show daily IgG EC50 titers for at least for 5 consecutive days within the range delimited by the 25th and 75th EC50 percentiles: i) low IgG: -log10(EC50) ≤ 25th perc (grey background); ii) medium IgG: 25th perc > -log10(EC50) ≤ 75th perc (blue background); iii) high IgG: -log10(EC50) > 75th perc (blue background). We define “day post-symptom onset" (PSO) as the day relative to the patient-reported onset of symptoms. (TIFF) [file pcbi.1009778.s001.tiff]

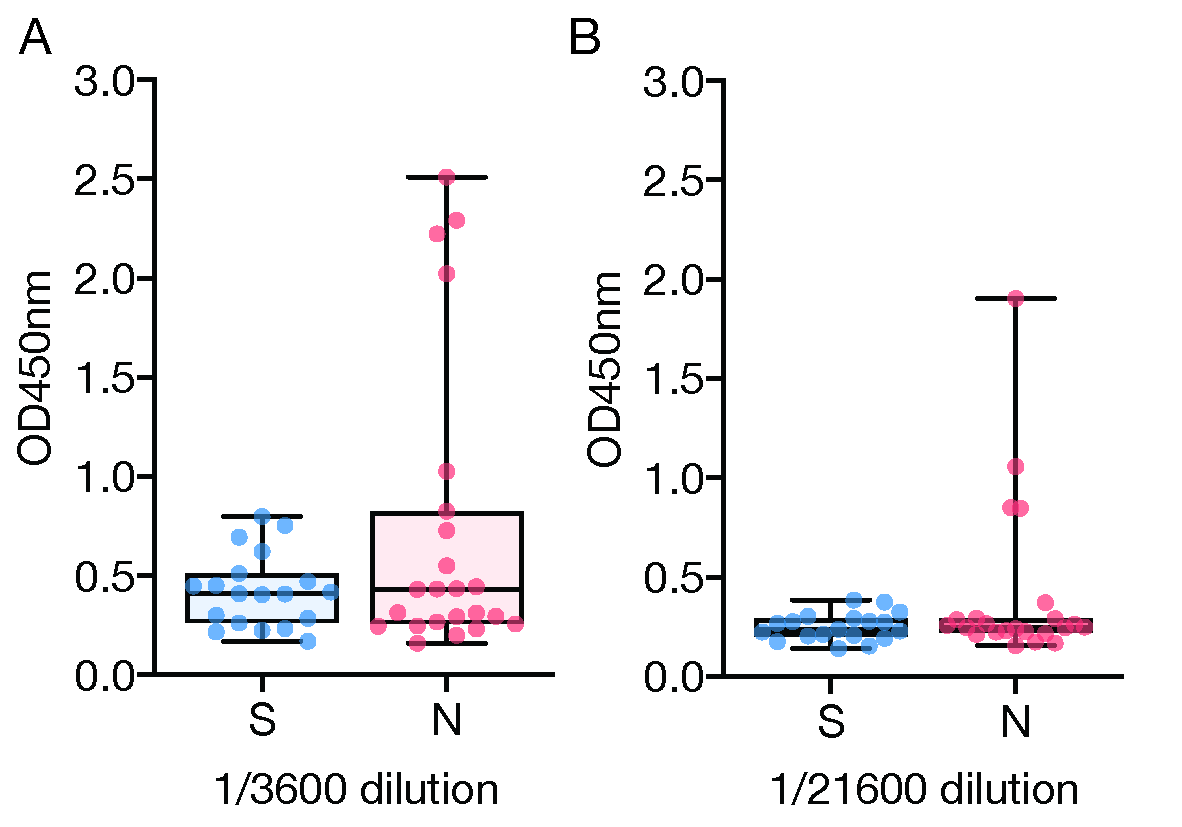

Supplement: S2 Fig — Comparison of IgG Ab response against SARS-CoV2 spike protein to nucleocapsid (N) protein. Anti-N and S IgG were measured at two serum dilutions and compared among hospitalized patients with a sustained low IgG response (patients = 17, serum samples = 23). ELISA experiments were performed in duplicates. Boxes extend from the 25th to 75th percentiles, the whiskers represent the minimum and maximum values and the middle line corresponds to the median. (TIFF) [file pcbi.1009778.s002.tiff]

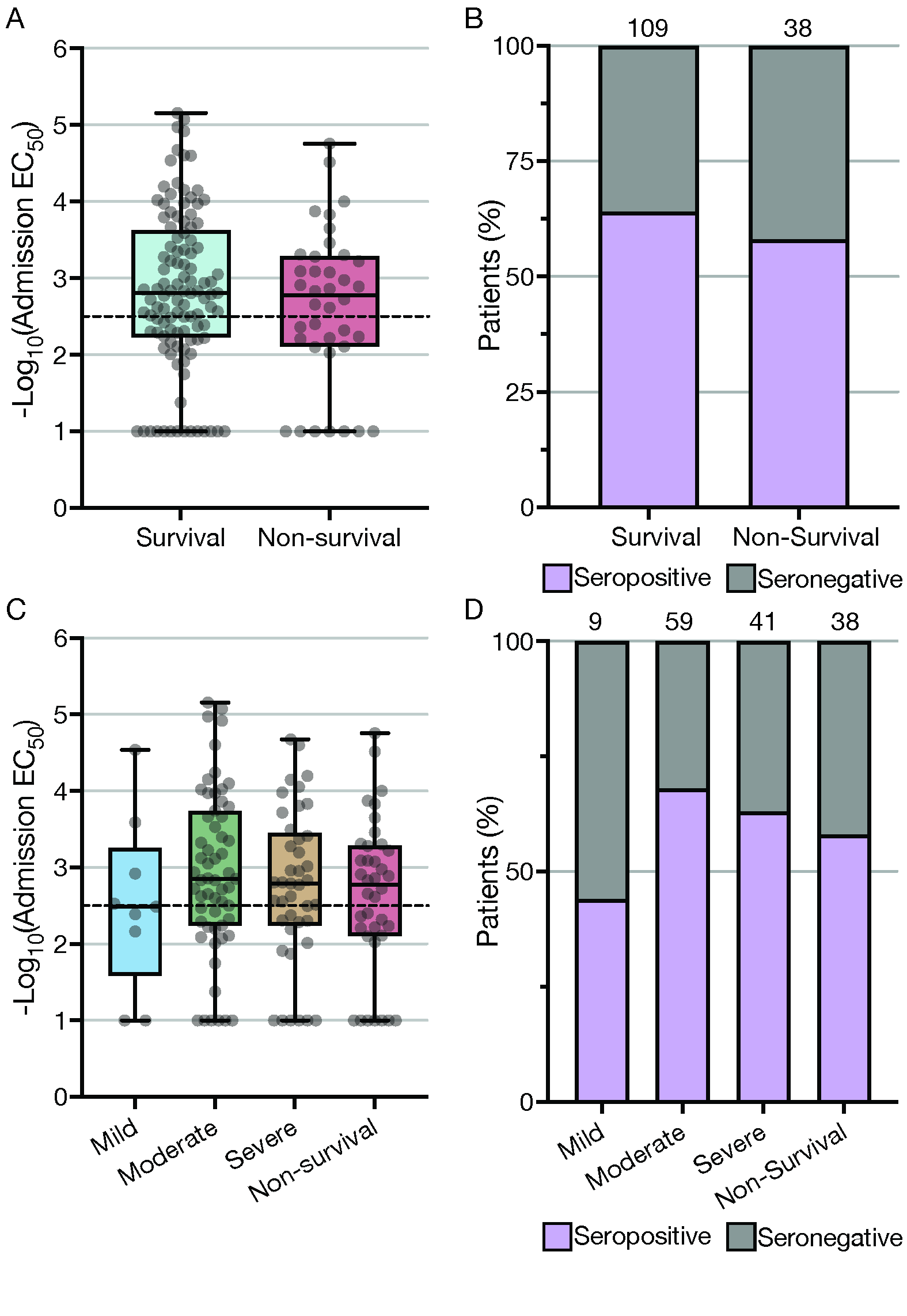

Supplement: S3 Fig — (A) Admission IgG titers for survivors (cyan) and non-survivors (red). No significance difference was observed between groups (Mann-Whitney p = 0.4). (B) Fraction of survivors and non-survivors that tested positive for antibodies (-log10EC50 > 2.5) the day of hospital admission (Purple: seropositive, Grey: seronegative). No significant association was observed (Chi-square p = 0.53). (C) Admission IgG titers for COVID-19 patients by clinical severity: mild (blue); moderate (green); severe (yellow) and; non-survivors (red). No statistical significance was observed (pairwise Dunn’s multiple comparisons p>0.05). (D) Fraction of patients by severity that tested positive for antibodies (-log10EC50 > 2.5) the day of hospital admission (Purple: seropositive, Grey: seronegative). No significant association was observed (Chi-square p = 0.51). (A, C) Boxes extend from the 25th to 75th percentiles, whiskers extend to the lowest and highest data point within 1.5 interquartile range of the lower and upper quartiles, the middle line corresponds to the median. IgG positivity threshold is indicated with a horizontal black dotted line at -log10(EC50) = 2.5. (TIFF) [file pcbi.1009778.s003.tiff]

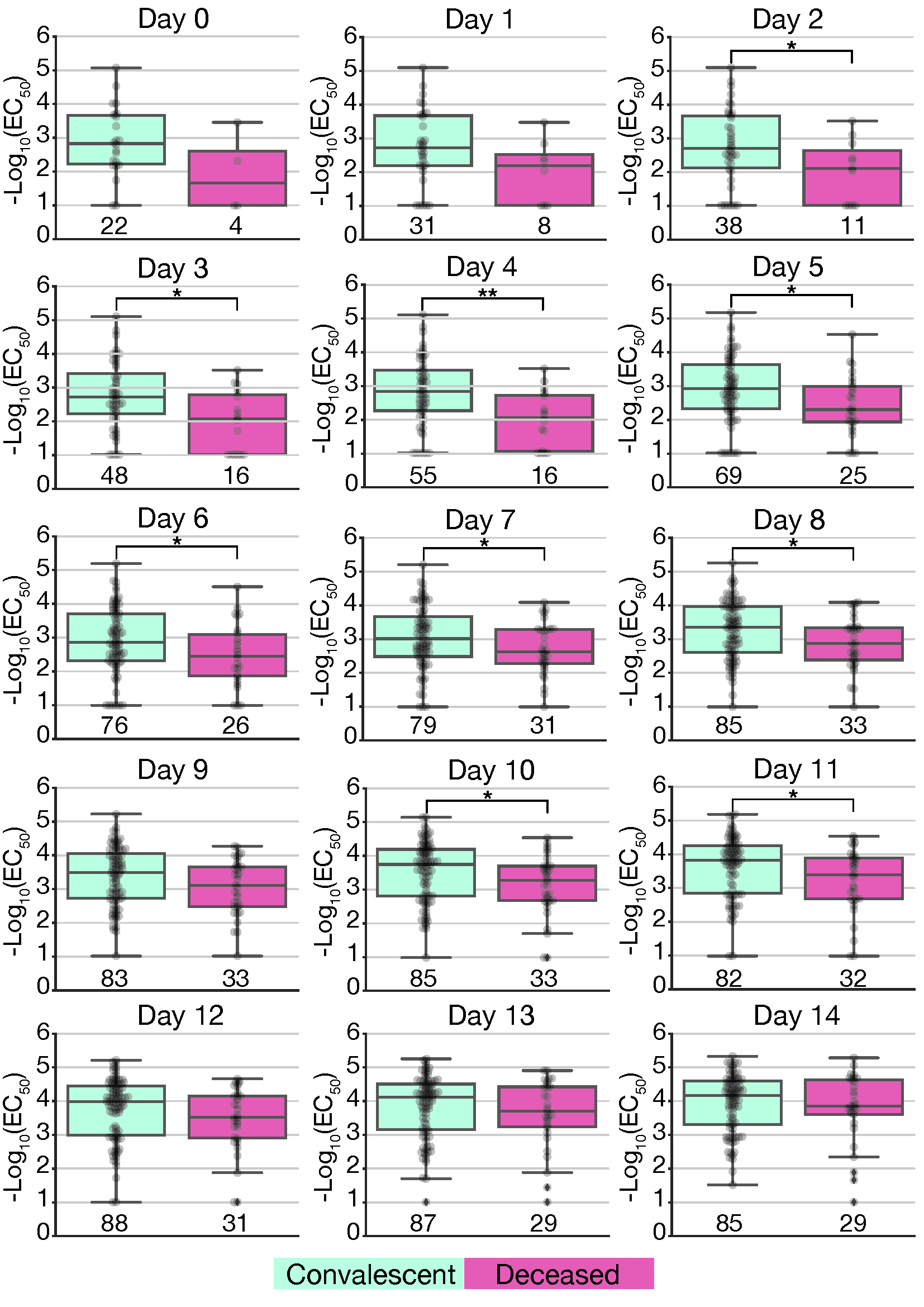

Supplement: S4 Fig — Box-plot at days 0–14 PSO comparing IgG titers, as dictated by the corresponding -log10(EC50), for survival (cyan) and non-survival (red) patients. Boxes extend from the 25th to 75th percentiles, whiskers extend to the lowest and highest data point within 1.5 interquartile range of the lower and upper quartiles and the middle line corresponds to the median. The size of each group is described under each boxplot. Statistical significance is denoted with asterisks (Mann-Whitney; *p < 0.05, **p < 0.01). We define “day post-symptom onset" (PSO) as the day relative to the patient-reported onset of symptoms. (TIFF) [file pcbi.1009778.s004.tiff]

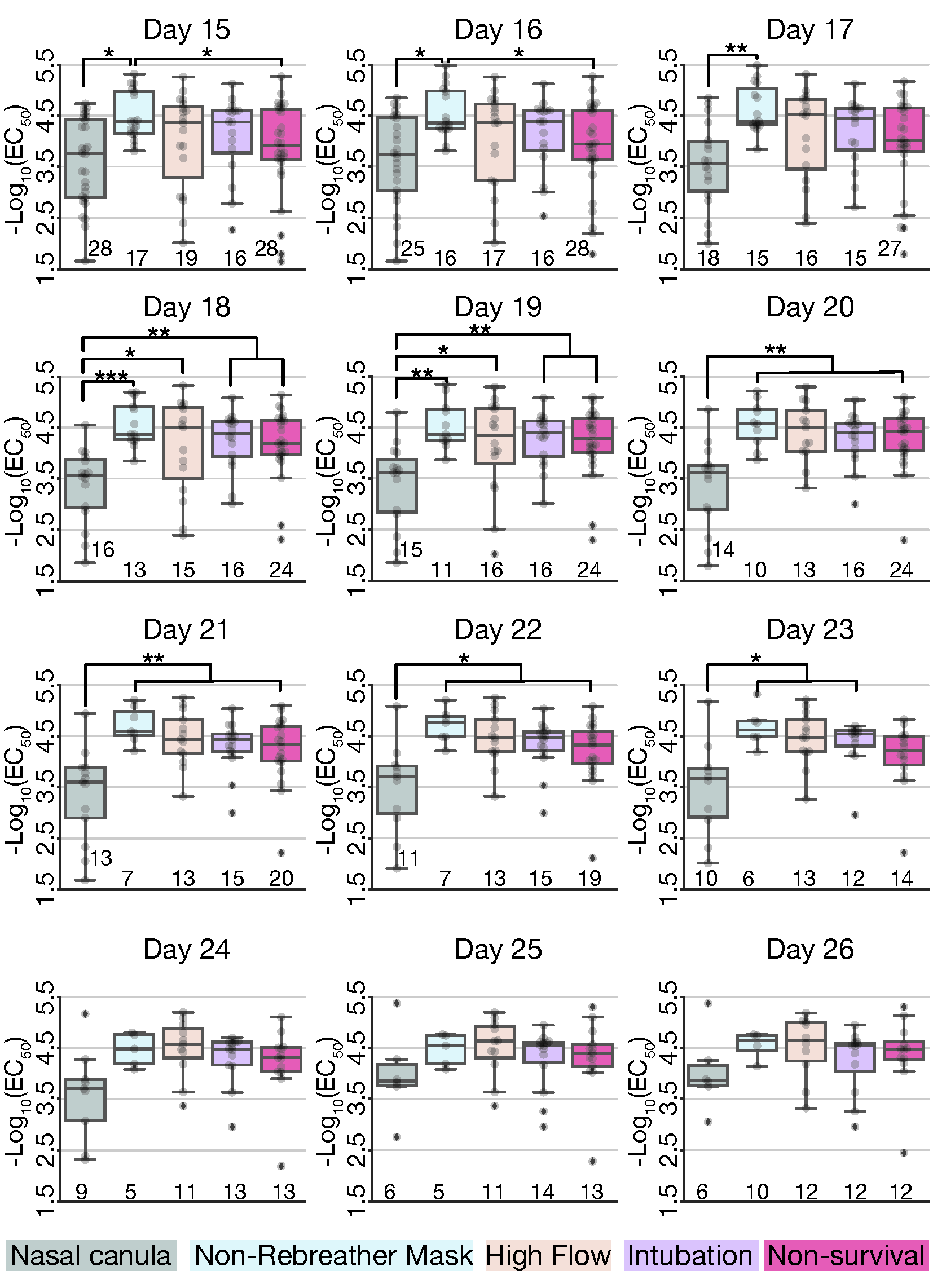

Supplement: S5 Fig — Box-plot at days 15–26 PSO associating IgG titers, by the -log10(EC50), by outcomes: surviving patients requiring maximal oxygen supplementation (nasal canula: grey, non-rebreather mask: blue; high-flow: yellow; intubation: pink) and non-survival patient (red). Boxes extend from the 25th to 75th percentiles, whiskers extend to the lowest and highest data point within 1.5 interquartile range of the lower and upper quartiles. The size of each group is described under each boxplot. Statistical significance is denoted with asterisks (Mann-Whitney; *p < 0.05, **p < 0.01). We define “day post-symptom onset" (PSO) as the day relative to the patient-reported onset of symptoms. (TIFF) [file pcbi.1009778.s005.tiff]

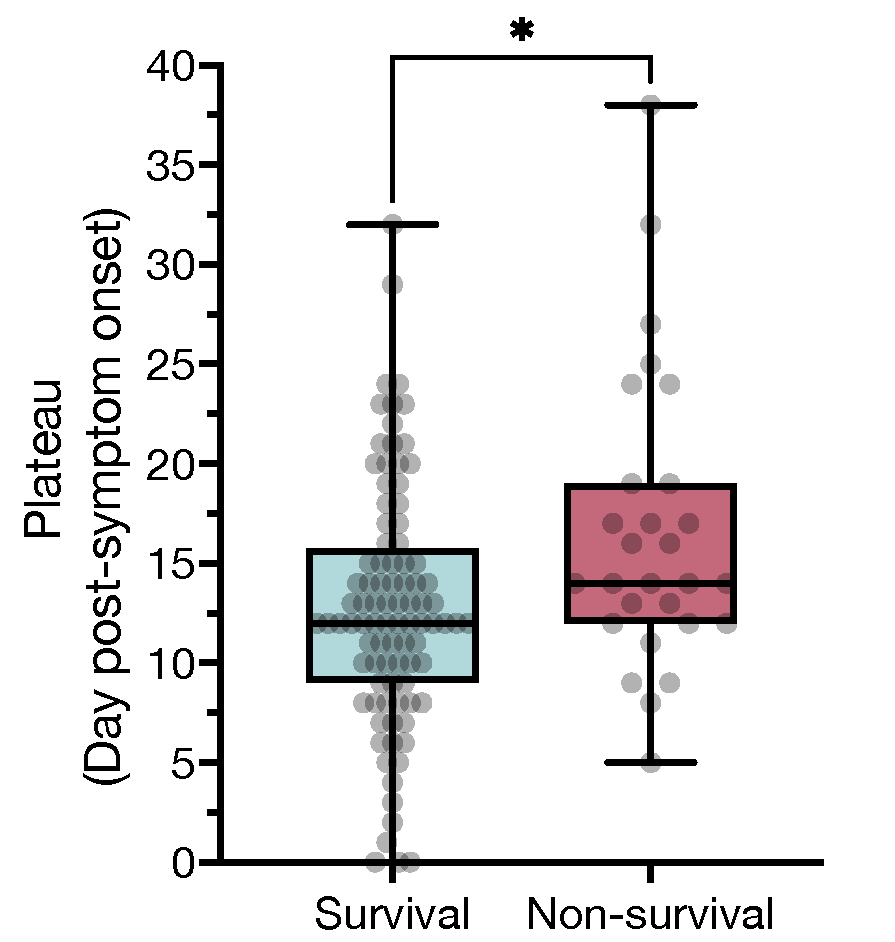

Supplement: S6 Fig — Day (PSO) at which IgG plateau is reached for survivors (cyan) and non-survivors (red). Plateau day for a particular patient is defined as the first day at which IgG titers reach 95% of the maximum IgG titer reported for that particular patient. Boxes extend from the 25th to 75th percentiles, whiskers extend to the lowest and highest data point within 1.5 interquartile range of the lower and upper quartiles, the middle line corresponds to the median. Statistical significance is denoted with asterisks (Mann-Whitney; *p < 0.05). (TIFF) [file pcbi.1009778.s006.tiff]

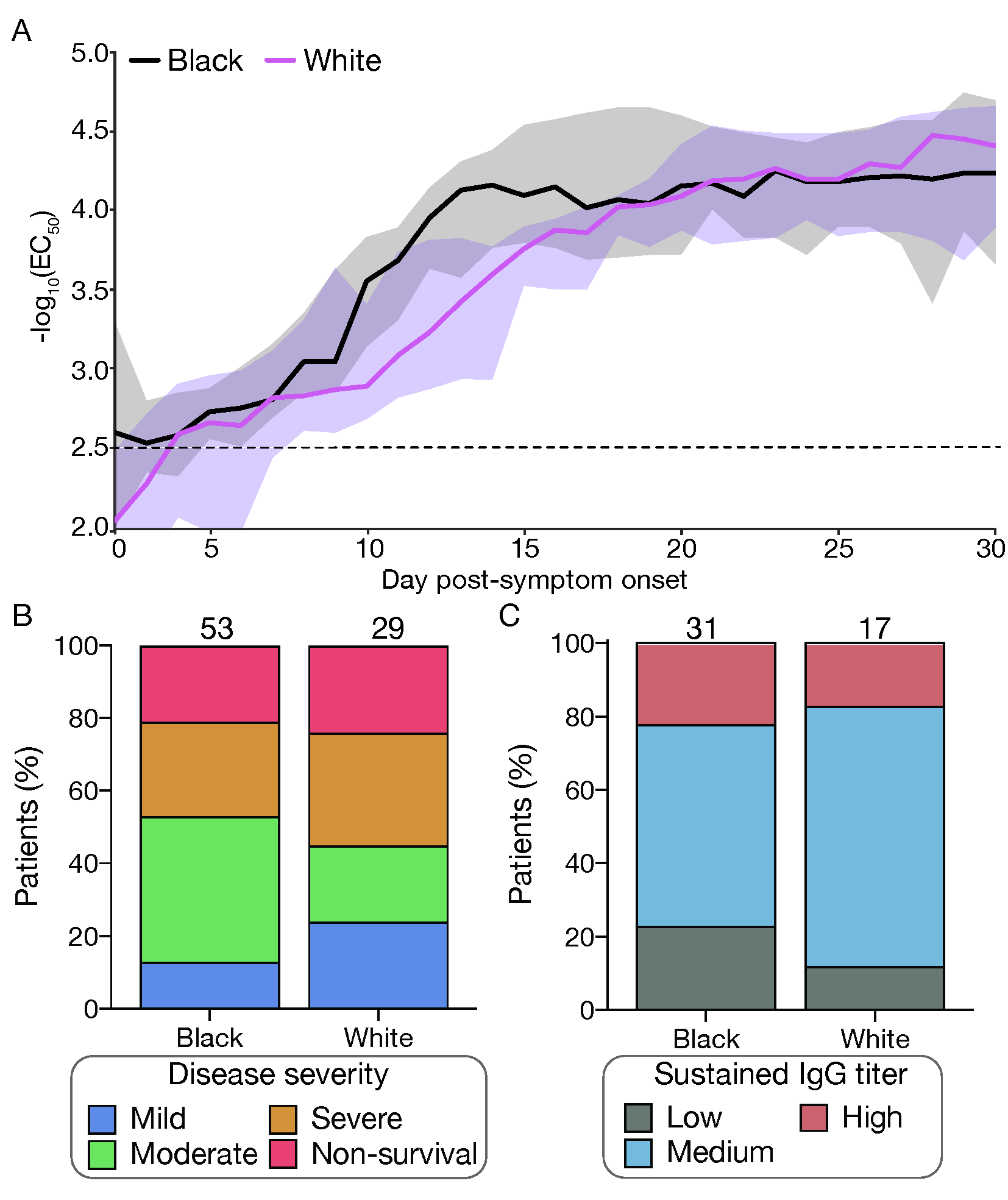

Supplement: S7 Fig — (A) IgG EC50 values (averaged value based on a five day sliding window) during the length of hospitalization of patients with different races: Black patients (black), White patients (pink). IgG positivity threshold is indicated with a horizontal black dotted line at -log10(EC50) = 2.5. Shaded areas correspond to 90% confidence intervals. We define “day post-symptom onset" (PSO) as the day relative to the patient-reported onset of symptoms. (B) Mosaic plot of COVID-19 disease (mild, moderate, severe, non-survival) relative to race (Black, White). (C) Mosaic plot of IgG sustainable responses relative to race (Black, White). There was no statistical differences between race and severity, mortality or sustained IgG response. (TIFF) [file pcbi.1009778.s007.tiff]

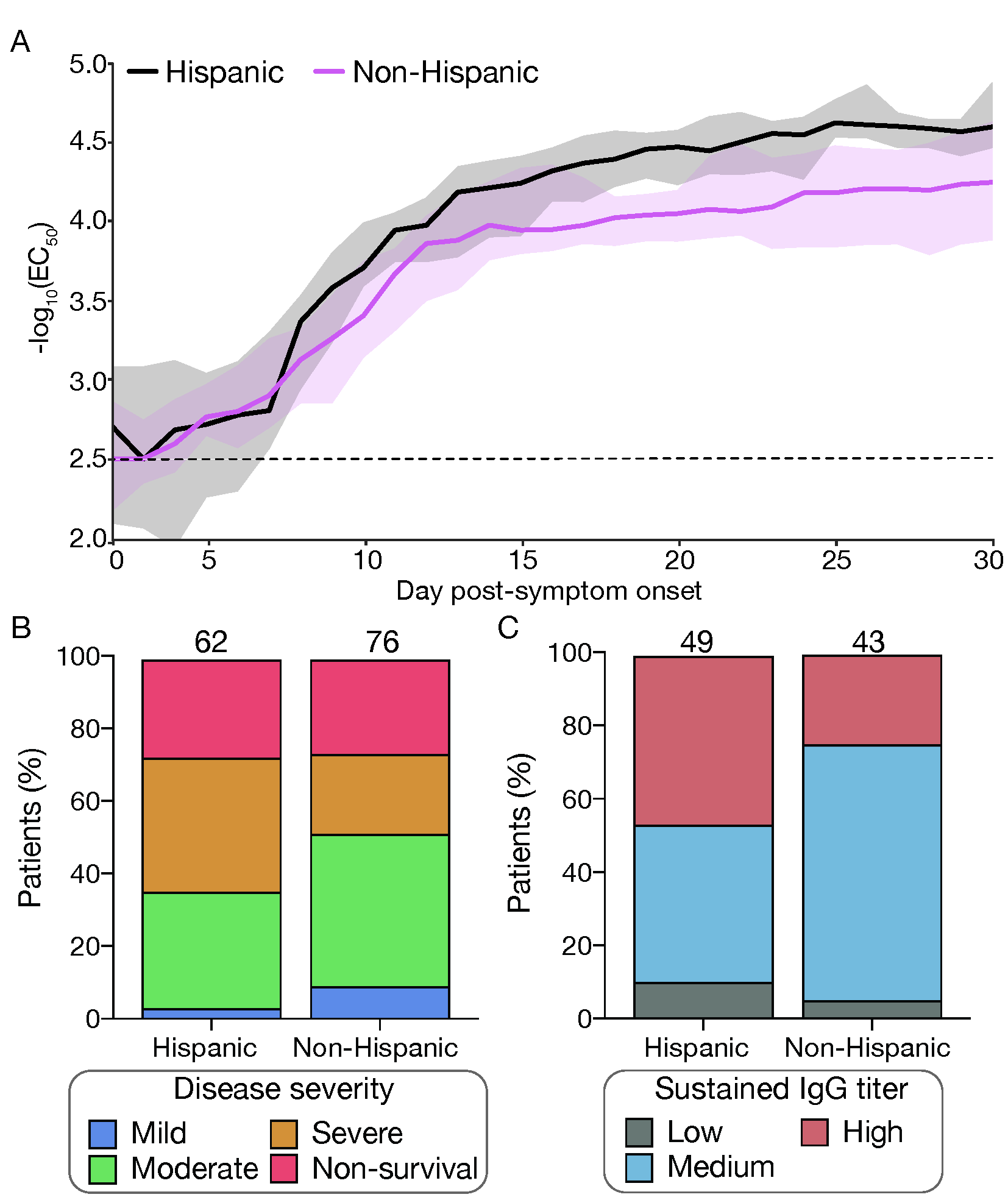

Supplement: S8 Fig — (A) IgG EC50 values (averaged value based on a five day sliding window) during the length of hospitalization of patients with different ethnicity: Hispanic and non-Hispanic (pink). IgG positivity threshold is indicated with a horizontal black dotted line at -log10(EC50) = 2.5. Shaded areas correspond to 90% confidence intervals. We define “day post-symptom onset" (PSO) as the day relative to the patient-reported onset of symptoms. (B) Mosaic plot of COVID-19 disease (mild, moderate, severe, non-survival) relative to Ethnicity (Hispanic, non-Hispanic). (C) Mosaic plot of IgG sustainable responses relative to Ethnicity (Hispanic, non-Hispanic). (TIFF) [file pcbi.1009778.s008.tiff]

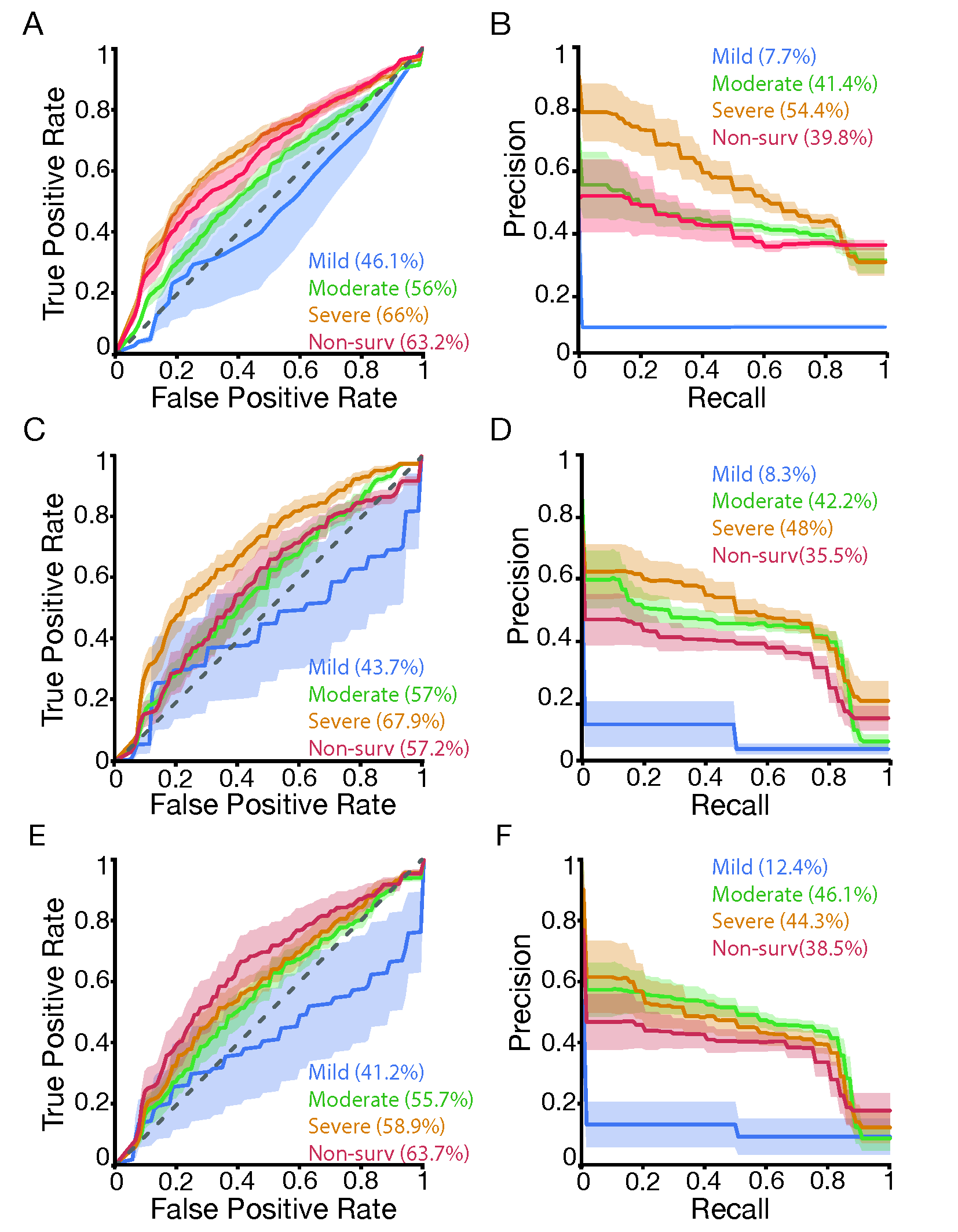

Supplement: S9 Fig — Roc and Precision-Recall curves resulting from the evaluation of (A-B) random forest, (C-D) logistic regression and (E-F) neural network to predict COVID-19 severity and mortality based on Electronic Medical Records (including clinical, other laboratory data and demographics; see S7 Fig at the day of admission. Shaded areas correspond to ± 2 standard error of the mean. Legends describe the corresponding area under the curve. (TIFF) [file pcbi.1009778.s009.tiff]

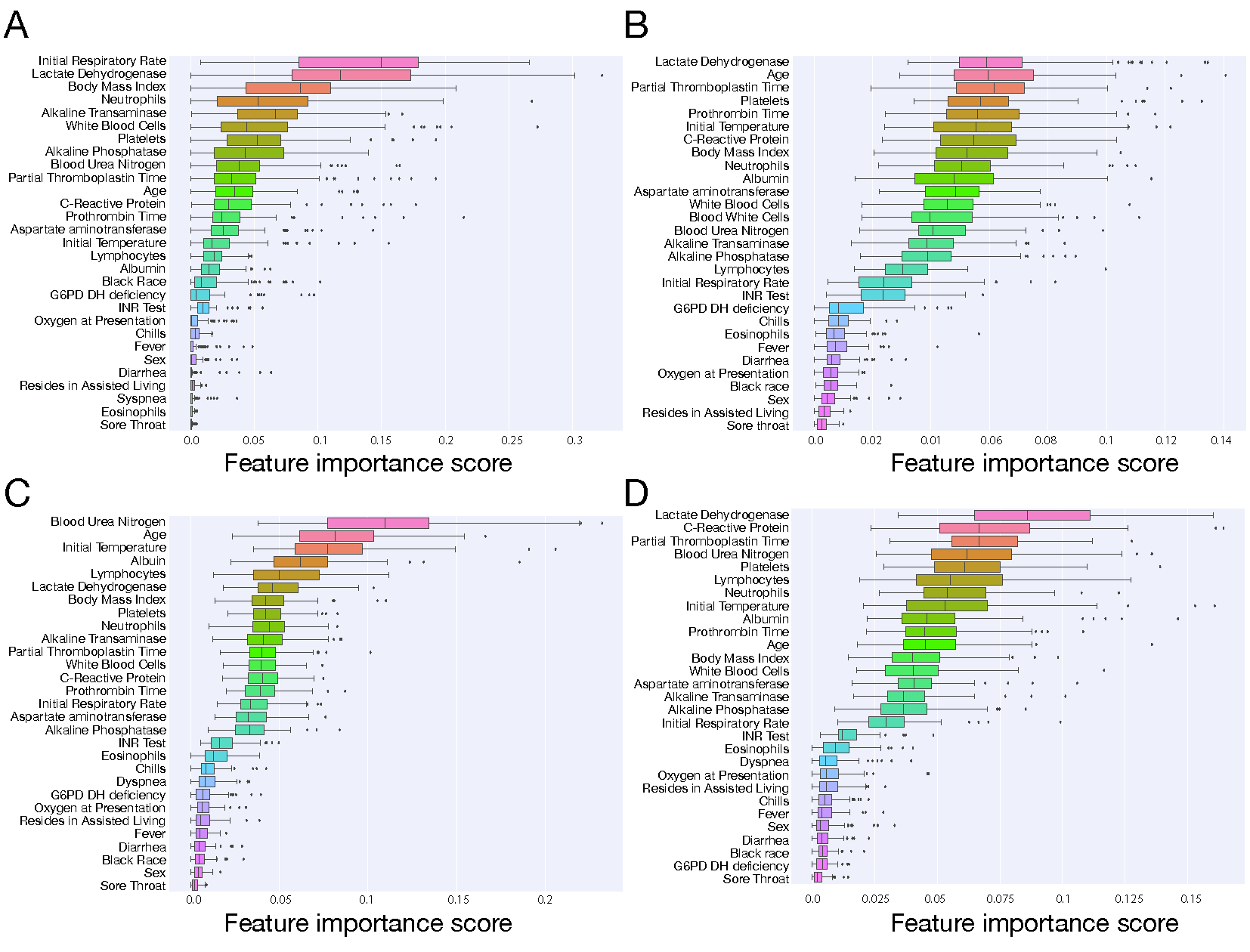

Supplement: S10 Fig — Features include clinical, other laboratory data and demographics. Each feature is ranked according to its corresponding importance score obtained by random forest to predict a particular category of disease, including non-survival: (A) mild; (B) moderate; (C) severe; and (D) non-survival. (TIFF) [file pcbi.1009778.s010.tiff]

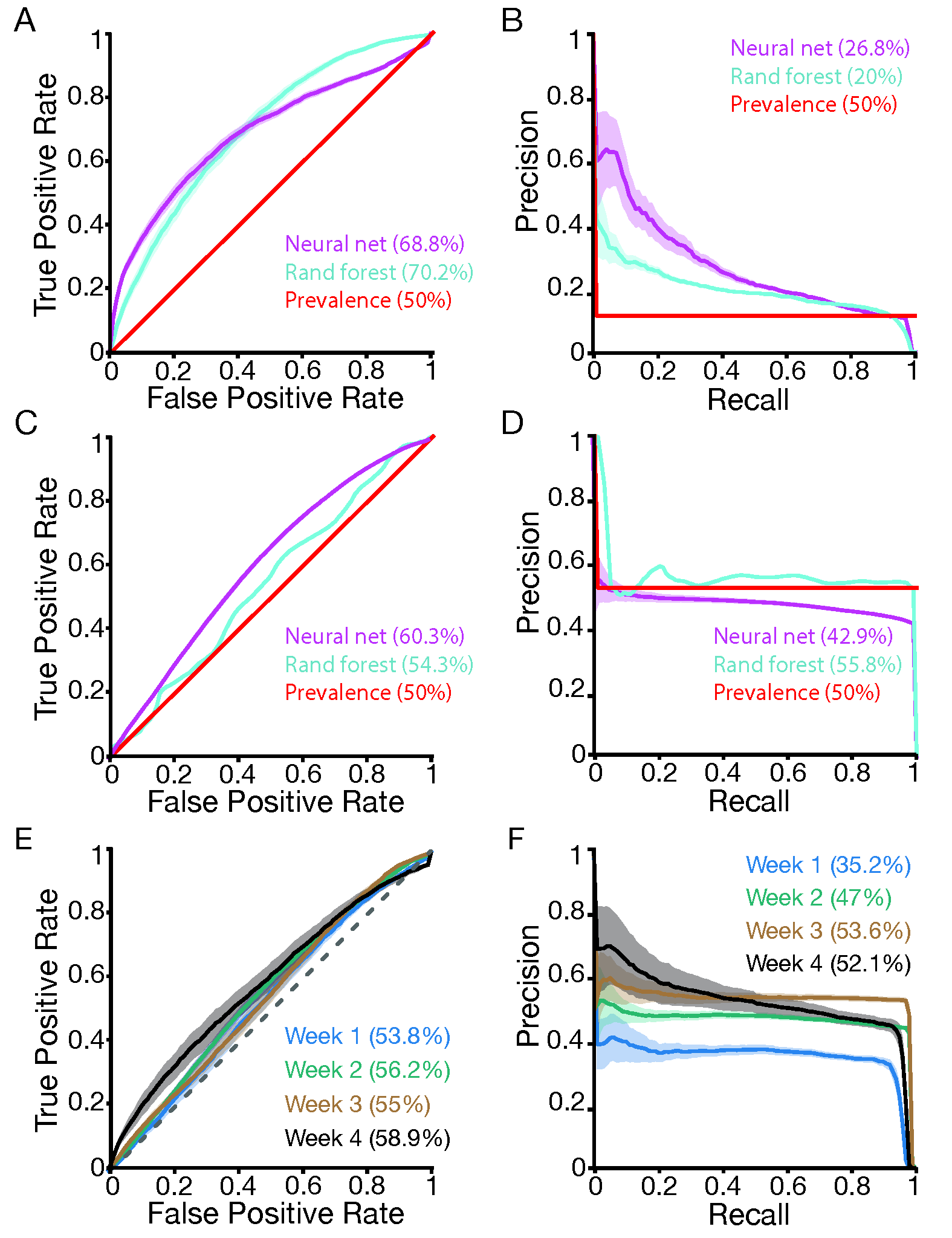

Supplement: S11 Fig — Evaluation of multiple machine learning methods to predict mortality and intubation using longitudinally-monitored clinical data. (A-B) Predicting mortality five days into the future; (C-D) Predicting intubation five days into the future (purple: neural network, dark blue: Logistic regression, light blue: Random forest, red: Prevalence). (E-F) Predicting intubation five days into the future at different hospitalization weeks using a neural network (green: week two, brown: week three, black: week four). Shaded areas correspond to ± 2 standard error of the mean. Legends described the corresponding area under the curve. (TIFF) [file pcbi.1009778.s011.tiff]

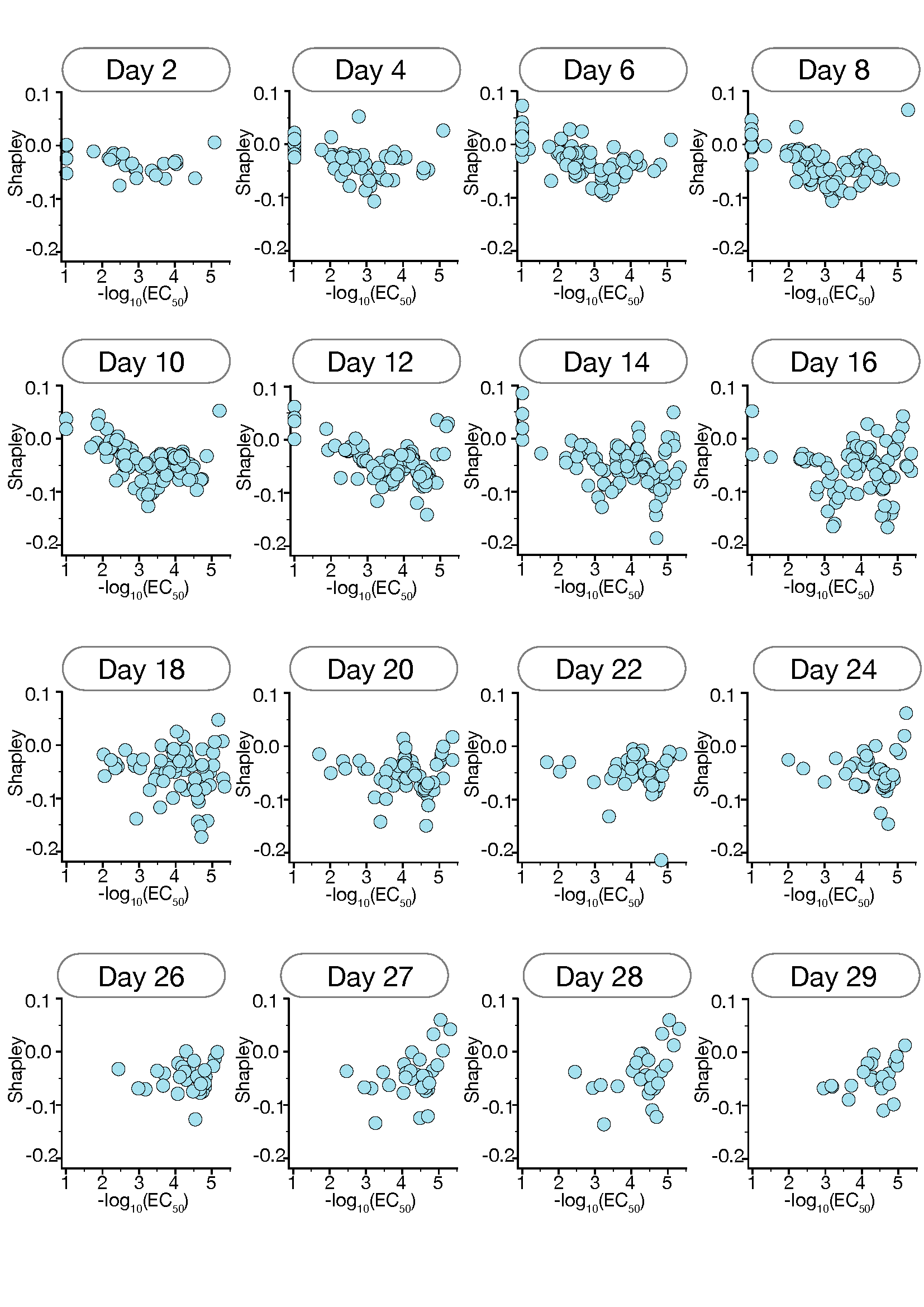

Supplement: S12 Fig — Plots describe the mean Shapley value Vs IgG titer at specific days PSO. We define “day post-symptom onset" (PSO) as the day relative to the patient-reported onset of symptoms. (TIFF) [file pcbi.1009778.s012.tiff]

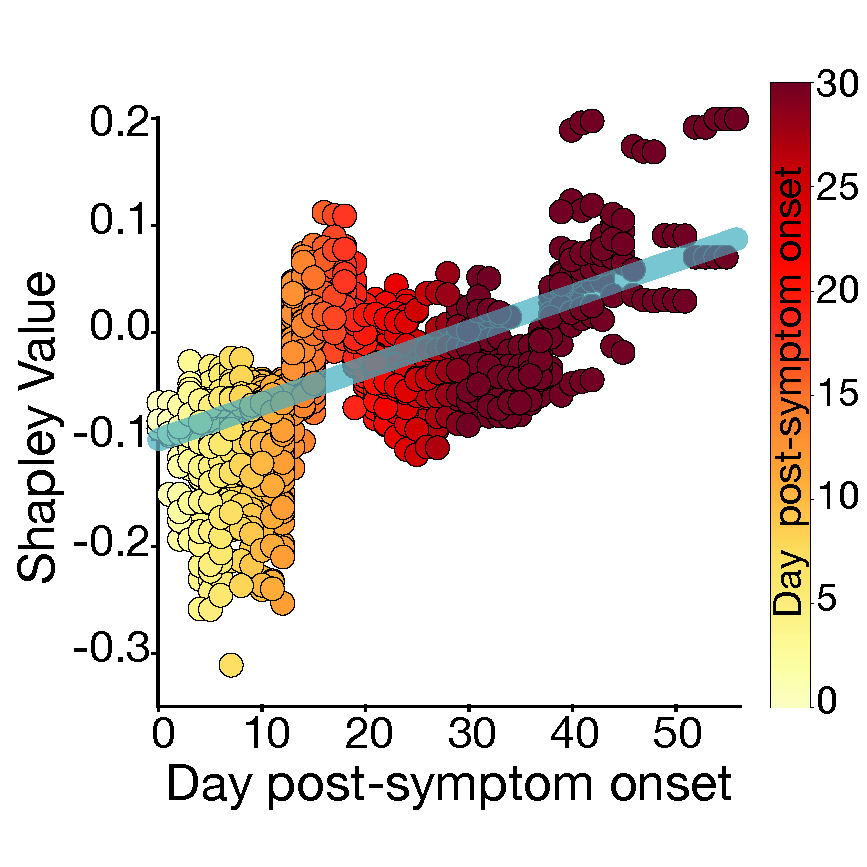

Supplement: S13 Fig — The plot shows the mean Shapley value compared to the day PSO for each sample in the dataset. We define “day PSO" (PSO) as the day relative to the patient-reported onset of symptoms. (TIFF) [file pcbi.1009778.s013.tiff]

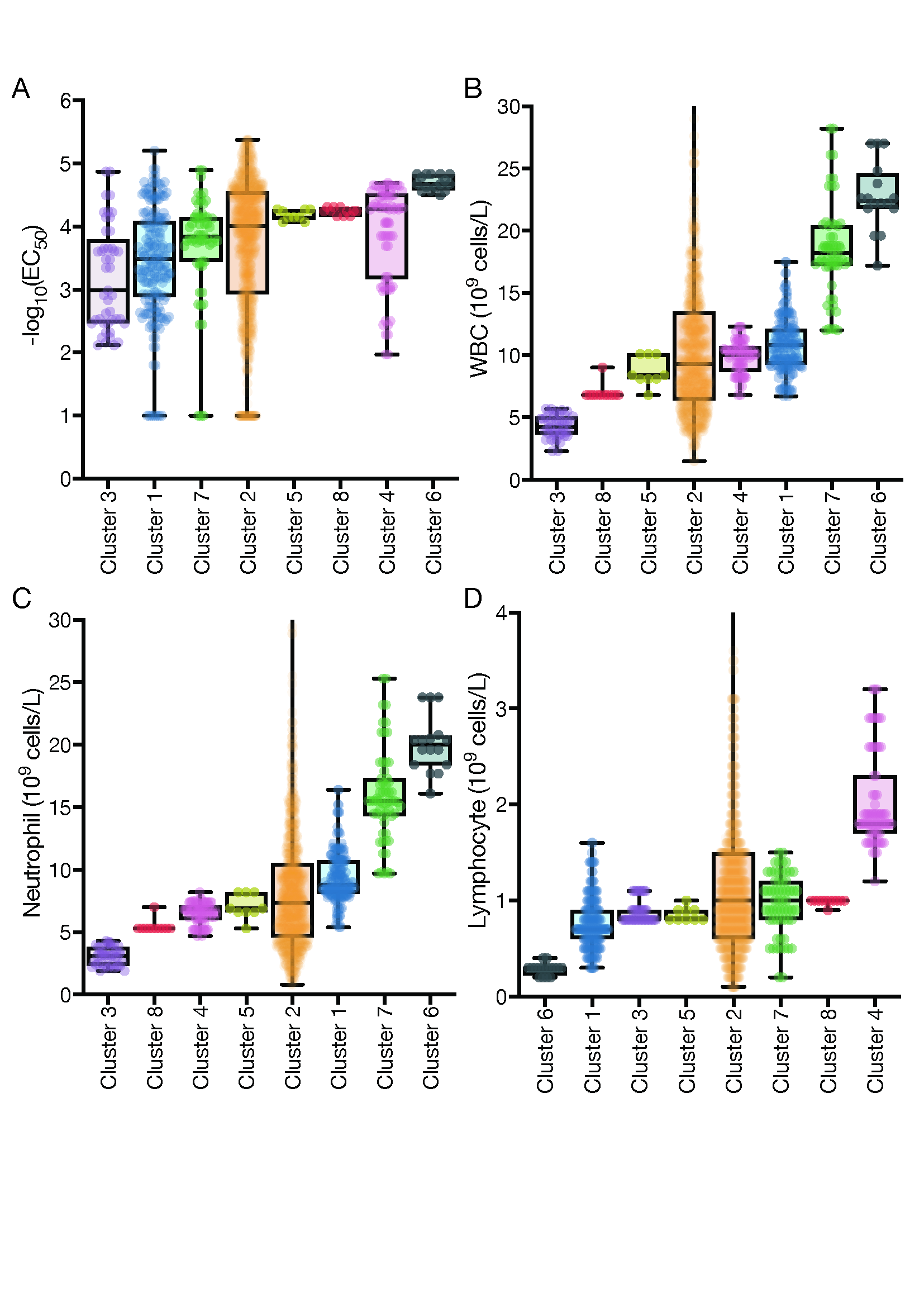

Supplement: S14 Fig — Comparison of clusters corresponding to patient samples obtained at any given day during hospitalization (see Fig 6E). Each single day of any given patient is defined by the corresponding Shapley values on immunological features monitored longitudinally. Boxes extend from the 25th to 75th percentiles, whiskers extend to the lowest and highest data point within 1.5 interquartile range of the lower and upper quartiles. (TIFF) [file pcbi.1009778.s014.tiff]
