# Supplementary material for: Longitudinally monitored immune biomarkers predict the timing of COVID-19 outcomes
Source: PLoS Comput Biol. 2022 Jan 18;18(1):e1009778. doi: 10.1371/journal.pcbi.1009778 (PMC8812869; doi:10.1371/journal.pcbi.1009778)
Supplement: S1 Table — Data are shown as number and percentage, n (%). Mild category is constituted of those who did not require supplemental oxygen. Moderate category is constituted of patients who required nasal canula (1-4L/min to maintain SpO2 >92%) or non-rebreather mask. Severe category is constituted of patients who were on non-invasive ventilation, high-flow oxygen (≥6L/min to maintain SpO2 >92%), invasive mechanical ventilation or extracorporeal membrane oxygenation (ECMO). Non-survival patients are those who deceased during the course of hospitalization. RR, respiratory rate, BP, blood pressure, AMS, altered mental status, PO, per os (given by mouth), AKI: acute kidney injury; ARDS: acute respiratory distress syndrome. (DOCX) [file pcbi.1009778.s015.docx]

|  | | **Total**  **(147)** | **Mild**  **(9)** | **Moderate**  **(59)** | **Severe**  **(41)** | **Non Survival**  **(38)** |
| --- | --- | --- | --- | --- | --- | --- |
| **Presenting symptoms** | | | | | | |
| RR no. (IQR) | | 20 (19-24) | 18 (17.5-19) | 20 (18.7-25) | 21 (18.5-25) | 21 (20-24.2) |
| BP systolic no. (IQR) | | 128 (115-144) | 130 (116.5-135) | 123 (111-140) | 128 (116.5-147.5) | 133.5 (122.5-146.3) |
| BP diastolic no. (IQR) | | 72 (63-80) | 70 (57.5-78.5) | 74 (65-80) | 73 (66.5-83) | 70.5 (60.75-77.5) |
| O2 sat total no. (IQR) | | 95 (92-98) | 98 (95.5-99.5) | 96 (93-99) | 94 (91.50-96.50) | 94 (90-96) |
| O2 sat (on O2) no. (IQR) | 95.5 (93—99) | | 96 (N/A) | 96 (93.5-99.75) | 95 (93-99.5) | 95 (92-99) |
| O2 sat (not on O2) no. (IQR) | | 95 (90-97) | 98 (95.25-99.75) | 96 (92-98) | 94 (88.5-96) | 92.5 (84.25-94) |
| AMS no. (%) | | 36 (24.5) | 1 (11.11) | 11 (18.6) | 11 (26.8) | 13 (34.2) |
| Given PO in first 24 hours no. (%) | | 141 (95.9) | 9 (100) | 57 (96.6) | 38 (92.7) | 37 (97.4) |
| **Signs and symptoms at admission**-no. (%) | | | | | | |
| Fever | | 106 (72.1) | 7 (66.7) | 39 (66.1) | 35 (85.4) | 25 (65.8) |
| Cough | | 95 (64.6) | 3 (33.3) | 34 (57.6) | 33 (80.5) | 25 (65.8) |
| Shortness of Breath | | 97 (66.0) | 4 (44.4) | 38 (64.4) | 28 (68.3) | 27 (71.1) |
| Fatigue | | 57 (38.8) | 0 (0) | 26 (44.1) | 17 (41.5) | 14 (36.8) |
| Sore Throat | | 8 (5.4) | 0 (0) | 2 (3.4) | 3 (7.3) | 3 (7.9) |
| Chills | | 27 (18.4) | 1 (11.1) | 8 (13.6) | 11 (26.8) | 7 (18.4 |
| Headache | | 18 (12.2) | 1 (11.1) | 8 (13.6) | 7 (17.1) | 2 (5.3) |
| Diarrhea | | 31 (21.1) | 3 (33.3) | 14 (23.7) | 8 (19.5) | 6 (1.6) |
| **Hospitalization complications-**no. (%) | | | | | | |
| AKI | | 79 (53.7) | 5 (55.6) | 24 (40.7) | 18 (43.9) | 32 (84.2) |
| Concurrent Pathogen Infection | | 48 (32.7) | 1 (11.1) | 13 (22.0) | 15 (36.6) | 19 (50.0) |
| Sepsis/Septic Shock | | 51 (34.7) | 2 (22.2) | 8 (13.6) | 14 (34.1) | 27 (71.1) |
| Cardiogenic Shock | | 4 (2.7) | 0 (0) | 0 (0) | 0 (0) | 4 (10.5) |
| Myocarditis | | 3 (2) | 0 (0) | 1 (1.7) | 0 (0) | 2 (5.3) |
| Cardiac Arrest | | 28 (19) | 0 (0) | 0 (0) | 0 (0) | 28 (73.7) |
| ARDS | | 52 (35.4) | 0 (0) | 0 (0) | 20 (48.8) | 32 (84.2) |
| ECMO | | 6 (4.1) | 0 (0) | 0 (0) | 1 (2.4) | 5 (13.2) |
