# Supplementary material for: Longitudinally monitored immune biomarkers predict the timing of COVID-19 outcomes
Source: PLoS Comput Biol. 2022 Jan 18;18(1):e1009778. doi: 10.1371/journal.pcbi.1009778 (PMC8812869; doi:10.1371/journal.pcbi.1009778)
Supplement: S3 File — (PDF) [file pcbi.1009778.s018.pdf]

Description of Models Used for 'Longitudinally Monitored Immune Biomarkers Predict the Timing of COVID-19 Outcomes'

Throughout this paper, all models were implemented using the Python package scikit-learn [1]. Plots associated with machine learning work were generated using the packages Seaborn [2] and Matplotlib [3]. All other plots were generated in R using the package Ggplot2 [4]. Plots were assembled in Adobe Illustrator.

1 Baseline Classifiers

Our baseline classifiers sought to predict the outcome of mortality or intubation from the input. We used admissions lab and intake values for this classifier. This included the following binary variables:
fever, shortness of breath, sore throat, chills, diarrhea, on o2 at presentation, black, male, residing in assisted living, and g6pd (indicating if patient had g6pd deficiency).
We used the following numeric values: age, bmi, initial temp, initial rr (respiratory rate), albumin, bunadmc (admissions BUN), neutadmac (admissions neutrophils), lymphadmac (admissions lymphocytes), astadmac (admissions AST), altadmac (admissions ALT), ldhadmac (admissions LDH), crpadmac (admissions CRP), inradmac (admissions INR), plateletsadminac (admissions platelets), pttadmac (admissions PTT), ptadmac (admissions PT), alkpadmac (admissiosn alkaline-phosphate), eosadmac (admissions eosinophils), wbcadminac (admissions WBC).
All values were as taken from the electronic medical records for those patients.
We evaluated three types of models: logistic regression, random forest, and neural network.

1.1 Models

1.1.1 Logistic Regression

We trained a logistic regression model using the L-BFGS method with a maximum iteration count set to 10000. During training, we used L^2 regularization with a regularization coefficient of .1.

1.1.2 Random Forest

We trained a random forest consisting of 50 trees trained using the Gini Impurity measure to determine tree splits.

1.1.3 Neural Network

We trained a neural network with one hidden layers consisting of 50 neurons. We used the ReLu (rectified linear-unit)activation function and trained the network using the Adam solver with a maximum of 1000 iterations. We also included a regularization parameter of .1.

2 Longitudinal Model

2.1 Dataset

We formulated the task of predicting future mortality of a patient as a sequence prediction problem: given all information up to time t, we seek to predict whether a desired outcome will occur within a time interval $(t, t+k]$ where k determines the window length. We model the trajectory of patient i as a sequence of tuples $(x_{i,t}, y^k_{i,t})_i$ where (x_t) and y^k_t correspond to the examples and labels at time t. $x_{i,t}$ is a vector consisting of the following (normalized) data points: -log EC50, Neutrophils, Lymphocytes, White Blood Cells, and day of sampling.

$y^k_{i,t}$ is determined as follows: Let $d_{i,t}$ represent whether or not a patient has passed away before time t. If patient i has passed away before or on time t, then $d_{i,t} = 1$, otherwise $d_{i,t} = 0$. We set $y^k_{i,t} = 1$ if any of $[d_{i,t}, d_{i,t+1}, ..., d_{i,t+k}]$ are nonzero (otherwise $y^k_{i,t} = 0$). In otherwords, it represents whether or not a patient will die within the next k days.

2.2 Model

The goal of our model is to predict $y^k_{i,t}$ given the history of past events $[x_{i,0}, x_{i,1}, ...x_{i,t}]$. Given the scale of training data available, we chose to simplify this problem and only consider data from the current time step. Hence, we seek to predict $y^k_{i,t}$ from $x_{i,t}$. To do this, we evaluated three types of classifiers: random forest, logistic regression, and neural network.

We performed 100 training runs for each model, each time randomly sampling a .75:.25 train/test split and randomly oversampling the minority class to balance the distribution of labels in the training set. We evaluated the performance of each model on its test set and used that information to generate receiver-operating characteristics and precision-recall curves. These curves were then averaged together to generate mean estimates and also provide an estimate

of the variance in our models. The drawn ROC curves represented the means plus-or-minus 2 standard errors of the mean.

Lastly, for the neural network models, we estimated Shapley values for each sample using the python package shap. In order to get a consistent estimate for Shapley values across training runs, we averaged Shapely values for each sample across each experiment that the sample showed up in the training set.

2.2.1 Logistic Regression

We trained a logistic regression model using the L-BFGS method with a maximum iteration count set to 10000. During training, we used L^2 regularization with a regularization coefficient of .1.

2.2.2 Random Forest

We trained a random forest consisting of 1000 trees trained using the Gini Impurity measure to determine tree splits.

2.2.3 Neural Network

We trained a neural network with two hidden layers consisting of 100 and 50 neurons respectively. We used the ReLu (rectified linear-unit)activation function and trained the network using the Adam solver with a maximum of 1000 iterations. We also included a regularization parameter of .5.

3 Background on Machine Learning Models

3.1 Logistic Regression

This is a model with performs binary classification by fitting the data to a logit function which takes values between 0 and 1. The logit function in our case is defined as $l_{i,t}^k = \log(y_{i,t}^k/(1-y_{i,t}^k))$. We perform a linear regression of $l_{i,t}^k$ on $x_{i,t}$ to get the following

$$l = \beta_0 + \beta_1 \cdot X$$

where $l, x \in \mathbb{R}^N \times \mathbb{R}^T$ are matrix representations of $l_{i,t}^k$ and $x_{i,t}$ across all N samples and T time points, and β_0 and β_1 are regression parameters [5]. There are a variety of methods for performing this regression in a statistically efficient manner. The method we used throughout this paper is called L-BFGS [6], which is a memory efficient adaptation of the Broyden-Fletcher-Goldfarb-Shanno algorithm which falls under the category of iterative methods. Briefly, the algorithm iteratively updates parameters based on a search direction which is informed by an estimate of the hessian of the objective function. This continues until a convergence criterion is met or a maximum number of iterations passes. Additionally, we use l^2 regularization to reduce the variance of our regression in exchange for biasing our parameter estimates towards 0 [5]. Essentially, this

means that we add a term $\lambda||\beta||_{l_2}$, where $||\cdot||_{l^2}$ is the l^2 norm, and β is a matrix concatenation of β_0 and β_1.

3.2 Random Forest

This model is an ensemble of decision tree classifiers [5]. The prediction of the model is determined by a majority vote of the predictions of the individual trees. Each tree is trained using an algorithm called CART (Classification and Regression Trees)[7] which iteratively splits trees into branches based on some information criteria. For our work, we used the Gini impurity index.

3.3 Neural Network

Neural networks consist of a sequence of layers, where the output of one layer x_i becomes the input to another layer x_{i+1} as follows:

$$x_{i+1} = \sigma(w_i \cdot x_i + \beta_i)$$

where w_i and β_i are model parameters which represent neurons within the network, and σ is an activation function which roughly binarizes its input [3]. In our work, we use the ReLu (rectified linear unit) activation function defined as:

$$\sigma(x) = \chi_{\{x>0\}} \cdot x$$

where χ is an indicator function.
We also train our network using the Adam optimizer [8]. This method falls under a family of optimization techniques based on gradient descent. Essentially, the optimizer moves in the opposite direction of the gradient of the objective function towards a local minima. In the case of Adam, there is an additional momentum term that enables the algorithm to avoid poor local minima. Like with the logistic regression, we also include l^2 regularization to reduce the variance of our models.

References

[1] F. Pedregosa, G. Varoquaux, A. Gramfort, V. Michel, B. Thirion, O. Grisel, M. Blondel, P. Prettenhofer, R. Weiss, V. Dubourg, J. Vanderplas, A. Pas-sos, D. Cournapeau, M. Brucher, M. Perrot, and E. Duchesnay. Scikit-learn: Machine learning in Python. *Journal of Machine Learning Research*, 12:2825–2830, 2011.

[2] Michael L. Waskom. seaborn: statistical data visualization. J*ournal of Open Source Software*, 6(60):3021, 2021.

[3] J. D. Hunter. Matplotlib: A 2d graphics environment. *Computing in Science & Engineering*, 9(3):90–95, 2007.

[4] Hadley Wickham. ggplot2: Elegant Graphics for Data Analysis. *Springer-Verlag* New York, 2016

[5] Trevor Hastie, Robert Tibshirani, and Jerome Friedman. The Elements of Statistical Learning. *Springer Series in Statistics.* Springer New York Inc., New York, NY, USA, 2001.

[6] Robert Malouf. A comparison of algorithms for maximum entropy parameter estimation. I*n Proceedings of the 6th Conference on Natural Language Learning* - Volume 20, COLING-02, page 1–7, USA, 2002. Association for Computational Linguistics.

[7] L. Breiman. Classification and Regression Trees. *CRC Press*, 2017.

[8] Diederik P. Kingma and Jimmy Ba. Adam: *A method for stochastic optimization*, 2017.
