# Supplementary material for: Longitudinally monitored immune biomarkers predict the timing of COVID-19 outcomes
Source: PLoS Comput Biol. 2022 Jan 18;18(1):e1009778. doi: 10.1371/journal.pcbi.1009778 (PMC8812869; doi:10.1371/journal.pcbi.1009778)
Supplement: S4 File — (DOCX) [file pcbi.1009778.s019.docx]

**Lymphocytes**

OLS Regression Results

========================================================================

Dep. Variable: Lymphocytes R-squared: 0.612

Model: OLS Adj. R-squared: 0.612

Method: Least Squares F-statistic: 3253.

Date: Wed, 17 Nov 2021 Prob (F-statistic): 0.00

Time: 15:59:47 Log-Likelihood: 2987.9

No. Observations: 2066 AIC: -5972.

Df Residuals: 2064 BIC: -5961.

Df Model: 1

Covariance Type: nonrobust

========================================================================

coef std err t P>|t| [0.025 0.975]

------------------------------------------------------------------------------

const 0.0693 0.002 29.962 0.000 0.065 0.074

x1 -0.0993 0.002 -57.038 0.000 -0.103 -0.096

========================================================================

Omnibus: 1080.458 Durbin-Watson: 0.566

Prob(Omnibus): 0.000 Jarque-Bera (JB): 22311.307

Skew: 1.996 Prob(JB): 0.00

Kurtosis: 18.596 Cond. No. 3.56

========================================================================

**Neutrophils**

OLS Regression Results

========================================================================

Dep. Variable: Neutrophils R-squared: 0.770

Model: OLS Adj. R-squared: 0.770

Method: Least Squares F-statistic: 6856.

Date: Wed, 17 Nov 2021 Prob (F-statistic): 0.00

Time: 15:59:47 Log-Likelihood: 3915.7

No. Observations: 2053 AIC: -7827.

Df Residuals: 2051 BIC: -7816.

Df Model: 1

Covariance Type: nonrobust

========================================================================

coef std err t P>|t| [0.025 0.975]

------------------------------------------------------------------------------

const -0.1653 0.002 -100.308 0.000 -0.169 -0.162

x1 0.0145 0.000 82.803 0.000 0.014 0.015

========================================================================

Omnibus: 163.143 Durbin-Watson: 0.719

Prob(Omnibus): 0.000 Jarque-Bera (JB): 490.827

Skew: -0.398 Prob(JB): 2.62e-107

Kurtosis: 5.259 Cond. No. 19.7

========================================================================

**WBC**

OLS Regression Results

========================================================================

Dep. Variable: WBC R-squared: 0.466

Model: OLS Adj. R-squared: 0.466

Method: Least Squares F-statistic: 1759.

Date: Wed, 17 Nov 2021 Prob (F-statistic): 7.29e-277

Time: 15:59:47 Log-Likelihood: 4300.6

No. Observations: 2018 AIC: -8597.

Df Residuals: 2016 BIC: -8586.

Df Model: 1

Covariance Type: nonrobust

========================================================================

coef std err t P>|t| [0.025 0.975]

------------------------------------------------------------------------------

const -0.0915 0.001 -66.233 0.000 -0.094 -0.089

x1 0.0049 0.000 41.935 0.000 0.005 0.005

========================================================================

Omnibus: 108.312 Durbin-Watson: 0.681

Prob(Omnibus): 0.000 Jarque-Bera (JB): 199.957

Skew: 0.395 Prob(JB): 3.80e-44

Kurtosis: 4.324 Cond. No. 25.5

========================================================================

**EC50**

OLS Regression Results

========================================================================

Dep. Variable: EC50 R-squared: 0.223

Model: OLS Adj. R-squared: 0.222

Method: Least Squares F-statistic: 296.0

Date: Wed, 17 Nov 2021 Prob (F-statistic): 9.24e-114

Time: 15:59:47 Log-Likelihood: 4305.5

No. Observations: 2071 AIC: -8605.

Df Residuals: 2068 BIC: -8588.

Df Model: 2

Covariance Type: nonrobust

========================================================================

coef std err t P>|t| [0.025 0.975]

------------------------------------------------------------------------------

const 0.0874 0.006 15.761 0.000 0.077 0.098

x1 -0.0746 0.003 -21.340 0.000 -0.081 -0.068

x2 0.0099 0.001 18.839 0.000 0.009 0.011

========================================================================

Omnibus: 197.634 Durbin-Watson: 0.542

Prob(Omnibus): 0.000 Jarque-Bera (JB): 522.344

Skew: -0.530 Prob(JB): 3.75e-114

Kurtosis: 5.221 Cond. No. 164.

========================================================================

**Day**

OLS Regression Results

========================================================================

Dep. Variable: Day R-squared: 0.296

Model: OLS Adj. R-squared: 0.296

Method: Least Squares F-statistic: 860.6

Date: Wed, 17 Nov 2021 Prob (F-statistic): 3.44e-158

Time: 15:59:47 Log-Likelihood: 3271.7

No. Observations: 2047 AIC: -6539.

Df Residuals: 2045 BIC: -6528.

Df Model: 1

Covariance Type: nonrobust

========================================================================

coef std err t P>|t| [0.025 0.975]

------------------------------------------------------------------------------

const -0.0990 0.002 -46.271 0.000 -0.103 -0.095

x1 0.0033 0.000 29.336 0.000 0.003 0.003

========================================================================

Omnibus: 11.873 Durbin-Watson: 0.444

Prob(Omnibus): 0.003 Jarque-Bera (JB): 12.034

Skew: -0.187 Prob(JB): 0.00244

Kurtosis: 2.961 Cond. No. 38.1

========================================================================
