# Supplementary material for: Longitudinally monitored immune biomarkers predict the timing of COVID-19 outcomes
Source: PLoS Comput Biol. 2022 Jan 18;18(1):e1009778. doi: 10.1371/journal.pcbi.1009778 (PMC8812869; doi:10.1371/journal.pcbi.1009778)
Supplement: Attachment — Submitted filename: Response_to_reviewers_9.docx [file pcbi.1009778.s020.docx]

Reviewer #1: The manuscript on longitudinally immune biomarkers predict the timing of COVID-19 outcomes addresses an important subject of death by organ failure with a respiratory virus.
The study utilizes a well characterized data set on 149 individuals hospitalized in New York from March 1 to June 1.

We would like to thank the reviewer for the constructive feedback and we appreciate the reviewer’s favorable opinion on our manuscript.

- The group has previously published on 103 patients given convalescent plasma from April to May 2020. <https://insight.jci.org/articles/view/142270>. Lacking from this paper is use of plasma, remdesivir and steroids which influences outcome in this population.

The reviewer is correct that some of the authors in the submitted manuscript participated in a study of convalescent plasma in hospitalized patients. The cohort in our current manuscript does not overlap with the JCI cohort and is from an earlier time point in the pandemic when convalescent plasma and remdesivir were not yet available. Consequently, none of the patients in our cohort received convalescent plasma, 15/147 patients received remdesivir, and 68/147 received steroids. A contingency test was performed to evaluate association between steroids and severity but no association was found. We more carefully describe these details.

- In table 1 traditionally comorbid conditions are compared between groups rather than within a group. Notably immunosuppression is 30% in mild, 15% in moderate, 12% in severe and 23% in nonsurvivors. Please add this analysis.

We agree with the reviewer and now table 1 and Supplemental Table 1 are updated to compare between groups rather than within a group. As requested, we have now in the immunosuppression category: 33.3% in mild, 15.3% in moderate, 12.2% in severe and 23.7% in nonsurvivors.

- Also under residency 113 and 35 =148 rather than 149 in total and 42 and 15=57 rather than 58 in moderate. Another typo is severe length of stay as median 19 with IQR 23 and 25. Might be 13 to 25??

We thank the reviewer for pointing out these typos. We corrected them in Table 1 and adjusted accordingly the percentage between groups. Also, please note that we removed 2 patients from our cohort who were not followed longitudinally during hospitalization and thus we have now 147 patients total.

- Another big point not explained is the spread amongst the groups of 130 analyzed for IGG which is the main point of the paper. Most likely more than half of the mild cases will be dropped. This reviewer would like to see the **numbers in the groups for the 130 in mild, moderate and severe and nonsurvivors. This could be added to Table 1**.

As suggested by the reviewer we have now included in Table 1 the number of patients falling into each IgG category: i) poor coverage due to insufficient data (see methods); ii) low,; iii) medium and; iv) high. Indeed 4 out of 9 patients with mild disease did not have sufficient IgG data to assess their prolonged IgG response. Out of the 5 remaining patients with mild disease, one patient was categorized as a low sustained IgG responder and four patients as medium IgG responders. Moreover, we have acknowledged in the discussion the benefits of including outpatients to better represent the mild disease category (**Lines: 464-466**): “*...although our dataset represents the hospitalized spectrum of COVID-19 disease, it would be interesting to also consider outpatients to be longitudinally monitored in a similar fashion*”.

- Another important point is the number of antibody negative patients in each group and what impact being antibody negative on admission has directly on the mortality.

Following the reviewer’s suggestions, we compared the IgG titers and seropositivity upon admission between: i) survivors and non-survivors and; ii) patients with variation in severity (mild, moderate, severe and non-survivors). In both cases we did not find statistical differences in IgG titers or serostatus between the different groups. We have included the results of this analysis as a new supplemental figure (Supp Figure 3) and updated the corresponding results section (**Lines: 222-223**): “*There was no association of hospital admission IgG titers or serostatus with disease severity (Supp Fig 3). However,...*”

- Another point that needs to be addressed is with exclusion of patients with less than 7 days of hospitalization most everyone is moderate to severe who is left for analysis. Mortality should be the single focus with so few mild cases in the analysis. For fig 2 F why only 77 cases and 109 in 2E. What was the reason for the dropouts.

We excluded patients who stayed less than 7 days in the hospital due to the lack of sufficient time points to assess their sustained IgG response. As suggested by the reviewer, Table 1 now describes the number of patients with poor coverage (and therefore excluded from this analysis) and patients with a sustained low (15 patients), medium (59 patients) or high IgG (35 patients) response. Please note that there were cases (21/130) where it was not possible to categorize the IgG response (despite being hospitalized for 7 days or more) since their IgG titers fluctuated between the established ranges. Those patients are described under the “Intermediate” category in Table 1. This has now been clarified in the results (**Lines: 182-184**) and methods section (**Lines: 574-576**).

We next assessed the potential association between the sustained IgG response (Low, Medium, High) and mortality, as suggested by the reviewer. The Chi^2^ test (p=0.24) showed no significant association. We added text to note that the small size of our dataset (15 patients with a sustained low IgG response) could limit this analysis. This analysis has also been described in the results section (Lines: 171-174)**:** “*We did not find a significant association between the category of sustained IgG response and mortality (Chi^2^ p=0.24). However, the lack of significance could be influenced by the small dataset (only 15 hospitalized patients showed a sustained low IgG response, where 46.7% of them died, Fig 2E)*”.

With respect to figure 2F, we are evaluating here the potential association between severity of symptoms and the sustained IgG response among survivors. Therefore, we discarded all deceased patients in this analysis. As the reviewer points out, the number of mild cases is small, thus we merged the mild and moderate categories into a single category to be compared against the severe category. To clarify the interpretation of this figure, we have updated the corresponding sentence in the results section (**Lines: 200-203)**, *“we found a strong association between severity of disease and the sustained IgG response categories (Chi^2^ p<0.01) among survivors…”*) and in the legend of figure 2F.

- The interesting finding was the low IGG titers in nonsurvivors at day 4 and then high at day 18 which is median day of death.

We agree that is a very important finding. The median day of death was 21 days, which we now more clearly report (**Lines: 162**).

- Another very confusing point is the switch back and forth of day form onset which is terrific to have but when used interchangeable with day of hospitalization. **This reviewer prefers both descriptors to be used for point of reference.**

We agree with the reviewer’s comment and have updated the figures and manuscript to report only day post symptom onset.

- **Can the authors attribute the increase in WBC and neutrophils to multiorgan failure versus pure respiratory death in Fig 4. What percent of nonsurvivors had multiorgan failure on day 18?**

Hypoxia (rather than multiorgan failure) was the cause of death in the vast majority of our study subjects (see Supp Table 1).

- The cluster analysis with Shapley values is an important aspect of the work. **Please note how it performs on Day 4 and day 18 which were previous benchmarks.** This was not clear from the discussion in result section and figure legends.

The reviewer has raised a valid and interesting point. Days 4 and 18 were chosen as representative for the early and late time windows based on IgG titers. The cluster analysis is based on IgG titers and neutrophil, WBC and lymphocyte counts and it is not meant to be predictive but rather to summarize our findings: certain clusters are enriched for either survival or non-survival and are associated with different levels of immune biomarkers, which aligns with statistical analyses described in the trajectories of specific biomarkers paragraph (**Lines 261-276**). In addition, we have added an additional supplementary figure (Supp Fig 12) describing the mean Shapley values of IgG titers at different days PSO (including days 4 and 18) and updated Figure 6 so that each datapoint is colored according to the corresponding day after onset of symptoms. In general, we observed that the relationship between IgG titer and the prediction of mortality gradually shifts from negative to positive as the number of days post-symptom onset increases. We have included this observation in the corresponding results (**Lines: 341-343**; “*Such relationship describes a gradual shift from negative to positive as the number of days increases (Supp Fig 12): lower IgG titers are more informative while predicting mortality early PSO and higher IgG titers are more informative when predicting mortality later PSO*”) and discussion (**Lines: 437-440**; ***“****For example, low IgG titers are more informative during the early severity time window to predict mortality (while high IgG titers are more informative days later PSO) and higher WBC and neutrophil counts are more predictive for fatal outcomes throughout hospitalization (Fig 6 and Supp Fig 12)****”***).

- Supplement table 1. Why was pulse left out of vitals?

Pulse was part of the patient electronic medical record (EMR) but this information was not extracted for each patient’s EMR and therefore cannot be reported. We have added in the discussion section a new paragraph acknowledging that additional features could also be considered (**Lines: 457**): “*Also capturing and analyzing day to day changes in oxygen supplementation and additional clinical and laboratory information (e.g. additional Ig titers) could provide more granular information to further inform pathogenesis models and treatment of severe COVID-1*9”.

- In fig 2 why were so few patients used in the first few days and more later? 22 to 85 ands 4 to 29 from day 0 to day 14

Some patients presented earlier in their disease; the numbers the reviewer references reflect the distribution of days post-symptom onset in our patients. We have clarified this in the corresponding results section (**Lines 143-145**): “*Patients were admitted into the hospital at different days relative to the onset of symptoms (median admission day PSO: 7; IQR 3-9) and had different lengths of stay (LOS) (median LOS: 13 days, IQR=9-21).*”

- Supplement fig 7- can admission IgG, or IgM be added to list

With respect to admission IgG we have addressed this question in an earlier comment made by the reviewer. Our statistical comparison (Supp Fig 3) showed that admission IgG levels do not differ between survivors and non-survivors and between patients with different degrees of severity (mild, moderate, severe and non-survivors). When including IgG titers in our random forest classifier the overall performance of the classifier decreases: the predictive performance decreases while predicting deceased patients (AUC decreases from 63.2% to 58.6%) and patients with moderate symptoms (AUC decreases from 56% to 51.5%) while it has no effect on predicting patients with severe symptoms (AUC 66%) and improves the prediction of patients with mild symptoms (AUC increases from 46.1% to 53.6%). Considering the overall lower performance when considering admission IgG titers, we decided to maintain our best performing models in Fig 5A and Supp Fig 7.

Regarding admission IgM titers: Unfortunately we do not have IgM titers on these patients. We have added a new paragraph in the discussion where we acknowledge that additional clinical features would likely inform our predictive models (**Lines 457-460**): “...*capturing and analyzing day to day changes in oxygen supplementation and additional clinical and laboratory information (e.g. additional Ig titers) could provide more granular information to further inform pathogenesis models and treatment of severe COVID-19.*”

- Please point out in figure 10 and in other results that the biomarkers are significant as a group but not in a single patient. Meaning that a patient with high IgG and high WBC may very well live.

We appreciate the reviewer’s comment. We have clarified our language to indicate that we are comparing groups of patients.

**Reviewer #2**: The authors use hospitalized COVID-19 patient data to predict disease outcome, in the form of oxygen support and mortality. Most importantly, longitudinal data during hospitalization (WBC, IgG titer, etc.) is used to make short-term predictions about mortality status, and the authors show that models trained on this data are more predictive that models that use only data from the time of admission. This is obviously an important study, and conceptually the approach makes sense to me. I do have one important suggestion to improve the analyses, and a bunch of small comments.

The "outcome" is also time-dependent. **Currently the most severe outcome is used, but surely non-survivors will be in the "severe" category before they die, and "severe" patients might be categorized as "moderate" at an earlier time point.** Can this additional information be used as well? This is most relevant in the longitudinal analysis, because the current disease status is not used as a predictor for the future status, while this is probably a very important predictor (see also my comment below). What kind of AUROC do you get if you include current disease status?

We thank the reviewer for their thoughtful analysis of our work, the suggestion that we consider current disease status in our models, and for their additional smaller comments.

Regarding use of a patient’s current disease status, the reviewer has raised an important point. Our categorization of patients into mild, moderate and severe classes is based uniquely on the type of oxygenation the patient received. To consider this feature in machine learning we would need to classify the severity status of each patient at any given day. Unfortunately, we do not have this detailed information; we only know whether a given patient received a particular oxygenation treatment during hospitalization but we do not know when it happened. The exception to this is intubation, for which we know when it began and ended. Thus we were able to include into our model the current intubation status as a predictor. We experimented with including this as a feature, but we found little to no impact on the resulting ROC metrics. This may be a limitation of the size of our dataset or the models we chose, but ultimately we excluded this from the main figure. We acknowledge the point raised by the reviewer, which we have brought up as a limitation of our study towards the end of the discussion (**Lines 457-460**).

Comments:

- Line 128: individuals with severe disease had a lower median age than moderate and non-survival. How can we explain the age difference between moderate and severe disease?

The severe patients also had a higher BMI (median 29.4) than patients with moderate disease (median 27.7) and this is one possible explanation for their higher severity of disease, despite their younger age. (See figure 1D)

- Line 143: one issue with the rolling five-day window is that you might include future data to make predictions. If you use observation X_t on day t, then X_t also contains information from days t+1 and t+2. This is relevant when you predict outcomes 1 and 2 days (and more) into the future.

We thank the reviewer for this comment. We used a rolling window to regularize data from our trajectories to correct for uncertainty in the time of sampling of immune titers. We numbered each hospitalization day based on the patient-reported onset of symptoms to evaluate clinical features under the same time frame. However, there is an uncertainty associated with the exact day when the first symptoms of illness appeared. The net effect of regularization is to minimize this potentially noisy information by describing data by the local mean, lowering the variance. Because the model is predicting mortality, not immune markers; if a patient dies, we don’t have immune titers for future time points .

- Line 149: Why did you choose unequal group sizes for this classification? Why not use 33th and 66th percentile?

Categorization of patients into sustained IgG response groups was based on the daily EC50 titers. We used the daily EC50 titers of all patients during hospitalization to set the thresholds (25^th^ percentile and 75^th^ percentile) that will delimit the ranges to classify patients. Therefore, the values used to assign thresholds (EC50) do not directly relate to the size of the groups (patients) but they play a role. For instance, using the 33^th^ and 66^th^ percentiles we do not have equal group sizes either: i) low sustained IgG response: 17 patients, medium sustained IgG response: 33 patients, high sustained IgG response: 50 patients. We have clarified this point in the corresponding methods section to better explain the methodology behind (**Lines 565-568**): “*Following this, all daily EC50 values (from all patients while hospitalized) were pooled together, the 25th (-log10(EC50) = 2.91) and 75th (-log10(EC50) = 4.47) percentiles were used as the lower and upper thresholds respectively to categorize their sustained IgG response of each patient.*”

- Line 155: 14.6% is the size of the Low group, right? So do you mean that a fraction of all patients had a Low response? That is because the groups are defined in this particular way.

Yes, 14.6% was corresponding to the size of the group of patients who had a Low sustained IgG titer for 5 or more consecutive days with a EC50 threshold set <25th percentile. Please note that based on reviewer 1 and your suggestions, we have added to Table 1 a bottom section called “sustained IgG” now describing the number of patients with poor coverage (and therefore excluded from this analysis) and patients with a sustained Low (15 patients or 11.5% instead of 14.6% as described before), Medium (59 patients) or High IgG (35 patients) response. We had some cases (21/130) where it was not possible to categorize the IgG response (despite being hospitalized for 7 days or more) since their IgG titers fluctuated between the established ranges. Those patients are described under the “Intermediate” category in Table 1. This has now been clarified in the results (**Lines: 198-199**) and methods section (**Lines: 560-576**).

- Line 157: The way the cutoffs are defined is a bit puzzling. As I understand it, you take all IgG titers from all patients pooled together, and then define 25 and 75th percentiles as cutoff for Low, Medium, High. Then the highest sustained (>5d) value per patient is used to assign a group to a patient. This can be explained a bit better.

The reviewer is correct on how we categorized the sustained IgG response. Based on the reviewer comments we have rewritten the methods section to clarify our procedure for classifying hospitalized patients and their sustained IgG response (**Lines 560-576**).

- Line 162: That is, only if the IgG response is observed before the outcome is observed.

We do agree with the comment made by the reviewer and we thank him/her for it. While we only “suggest” the potential role of IgG titers to predict COVID-19 severity, we felt more comfortable by removing this statement from the text. The potential role of IgG titers to predict mortality is addressed in **lines 221-232 & 339-346** of the manuscript. .

- Line 171: One small remark: Because the maximum IgG per patient is used, those that stay in the hospital longer also have a higher chance of falling in the High category. So if the IgG titers were just random, you would find the same result. Of course they're not random, but perhaps you could test if the result still holds of you use the mean titer instead of the maximum.

We thank the reviewer for this comment. Our results as described in figures 2D and 3A show that IgG levels tend to increase within the first three weeks after onset of symptoms and then remain stable throughout the length of hospitalization. This argues against the hypothesis that longer hospitalization will lead to higher IgG titers. To further illustrate IgG trajectories, we have included an additional supplementary figure (Supp Figure 1) plotting individual IgG trajectories of patients and their corresponding sustained immune response (Low, Medium, or High). The corresponding results section has also been updated to describe this observation.

With respect to using the mean instead of the maximum IgG titers, we don’t describe the patient’s trajectory by their maximum IgG titer. Our script (available in the GitHub repository) uses all the single daily titers, and classifies each patient’s trajectory based on the highest sustained (at least five consecutive days -so at least five consecutive IgG titers-) IgG titer that extends to the end of the hospitalization stay. We apologize for any confusion our text might have led to. We have rewritten the corresponding method section in order to better explain this (**Lines 560-576**).

- Line 193: I don't quite understand the statement starting with "consequently". Why are IgG titers not associated with both severity AND mortality?

The reviewer is correct, high IgG titers in the late time window are associated with both severity and mortality, we have corrected this (**Lines 228-229**)

- Line 196: refer to a figure. Is there a statistical analysis that confirms this?

We have included a new supplemental boxplot figure describing the significance of the difference (Supp Figure S6). The result of the statistical analysis has also been reported in the line the reviewer refers to (**Lines 239-240**): *“non-survivors tend to reach a plateau later than survivors (IQR 9-16 days and 12-19 days respectively; Mann-Whitney p= 1.2x10^-2^, Fig 3A and Supp Fig 6)”*

- Fig 4: WBC and Neutrophil for non survivors have a very specific shape (increase followed by decrease). Is there a relation with the times that patients died? Would it be possible indicate these times in the plot?

Following the reviewer’s request, we have specified the time of death in main figures 3A and 4. Figure legends have been updated. During the first 25 days after onset of symptoms (which roughly speaking divides the WBC and Neutrophil trajectories into an ascending and a descending half) 64% of the non-survivors died. 35.9% of the non-survivors died between days 19-23, which overlaps with the end of the ascending trajectory and the beginning of the plateau of max cell counts.

- Line 237: A bit more words could be added to explain what the models do: is each model trained on one particular binary outcome? (moderate or non-moderate disease? mortality or survival?).

We agree that we need to provide more clarity into how the models function and are trained. We have added a supplemental methods section describing how each model works in more detail (Supp file 3).

- Line 265: Here you only mention mortality status, but later you also predict intubation status.

We thank the reviewer for this observation, the corresponding text has been updated to clarify this issue (**Lines 316-318**): “*...we implemented machine learning models to predict a patients’ mortality status (Fig 5B-D) and intubation (Supp Fig 11) within the next k days using immune biomarkers that would have been available on a given day .*”

- Figure 5B: the predictive performance increases with time since hospitalization (is that what you mean by length of hospitalization?). Is this because during the first week not many patients died, and hence there is nothing to predict?

The predictive performance increases with day post-symptom onset, not with length of hospitalization, we apologize for the confusion. The model considers time post-onset of symptoms rather than time since hospitalization. We made this adjustment based on reviewer comments to align with the rest of the analyses in the paper which are all conducted relative to onset of symptoms. Also we have clarified Fig 5 legend accordingly.

Several lines of evidence suggest that our observation that predictive performance increases over time is a real phenomenon. First, ROC curves are a function of sensitivity and specificity of a classifier as a threshold is varied. These are invariant to the distribution of true and false labels in a dataset (in this case, that would be the number of patients who died vs did not die in a week). Hence, we cannot explain the low ROC performance in the early periods simply from an imbalance in mortality. Precision recall curves are, on the other hand, affected by class imbalances. As figure Supp 11F shows, the precision recall in the earlier weeks is lower than the later weeks as a result of less positive samples to predict. One caveat is that all of these curves are statistical estimates based on sampled data. As a result, there is noise in the estimation. We mitigated some of this noise by averaging metrics across multiple runs, but when the training data is small, that noise is always inherently greater. As the reviewer notes, we have a smaller number of samples during the first week. We analyzed the relationship between the number of patients for a given day vs the average AUC and found no correlation. However, the variability in the average AUC does become higher as we have less patients on a given day.

- Line 268: Have you also tried including the current disease status as a predictor? I can imagine that this could be significant, and it is also easily available.

We agree that this is an important point; we have responded above.

- Line 282: For which analysis are you doing this? The longitudinal or static data? The quadratic relation can be understood given that early, low IgG is associated with mortality, but also high late IgG. Is it possible to plot this together with the day of measurement to confirm this? Perhaps color the dots to indicate time?

We conducted the Shapley value analysis on the longitudinal (neural network) model. The sub header of the section has been updated to clarify this (**Lines 332-333**): “*Shapley Values on the Longitudinal Model Identifies Patient Clusters with Higher Likelihood of Outcomes*”.

We absolutely agree with the reviewer with respect to the relevance of low and high IgG titers relative to the onset of symptoms. Following the reviewer’s suggestion, we updated our Shapley value figures (Fig 6 A-D & Supp Fig 13) to color code by day. The resulting plots indeed show a color gradation, highlighting that the relationship between IgG and prediction of mortality flips as the disease course progresses. To further highlight this, we have also included an additional supplementary figure (Supp Fig 12) where shapley plots are shown for different days PSO. As described in the results section (**Lines 341-343**): *“Such relationship describes a gradual shift from negative to positive as the number of days increases…”*.

- Line 468: The information here requires that the reader has experience with these packages. Is there a way to explain the settings in a more general way? For instance, what does regularization alpha of .1 do?

We have put together a more detailed description of our methods attached as a supplemental file 4. The supplemental methods explain the formal model description in more detail and describes the parameters used in the models.

- Also, I think that you should definitely publish your scripts (on github or a similar platform), because it should be easy for others to repeat these analysis with their own data.

We would like to thank the reviewer for bringing up this important matter. At the time of submission we did include our GitHub repository but for reasons we do not understand the reviewer was not able to see the link. The scripts have been made publicly available on the GitHub repository. We have also included a link to the GitHub repository in the corresponding method sections (**Lines 663-664**): “*Scripts, datasets and results are available at our GitHub repository:* [*https://github.com/kellylab/longitudinal-prediction-of-covid-outcomes*](https://github.com/kellylab/longitudinal-prediction-of-covid-outcomes)”

**Reviewer #3**: Overall comments:
COVID-19 data suggests an intrinsic relationship between disease progression, severity of symptoms and immune markers. In this work, the authors measure longitudinally immune biomarkers of 147 hospitalised COVID-19 patients (e.g. neutrophils, lymphocytes, IFN). By examining their hospitalised patient measurements, they find survivors have a higher basal IgG titer at the day of hospital admission, compared to non-survivors. Patients who died during their hospitalisation had much lower IgG titers from days 2-11 compared to survivors. In addition, they find a clear delineation between survivors and non-survivors based on the longitundinal measurements of lymphocytes, neutrophils, and white blood cells. These results are not surprising as there are other studies of longitudinal data that echo results, however, it is still of interest to see these results confirmed in this study.
To investigate the relationship between biomarkers and fatality, the authors developed a machine learning framework trained on the longitudinally monitored immune biomarkers of hospitalised patients. They show that the framework can predict mortality more accurately than demographic and clinical data obtained upon hospital admission, i.e. using longitudinal measurements predicts mortality more accurately than models using single initial hospitalisation measurements. This also doesn’t feel surprising, as it suggests we can make more accurate predications with more data. While the work in this manuscript is very interesting, I feel the authors have a few areas that need addressing. I’ve detailed my comments/queries below.

Major

- In Fig 1A, it is not clear to me from the caption or the reference in the text why there are multiple data points for an individual patient corresponding to the yaxis label “Hospitalisation day after symptom onset”. Also, what are the clinical features that are colouring the heatmap? Is it disease severity or something else? The colour scheme also makes it very difficult to determine which patients experienced certain clinical features at certain times, i.e. to see the light red vs the dark red. I think this figure needs to be improved for clarity and needs more explanation. I would have thought that “hospitalisation day after symptom onset” should be a single measurement or data point I assume as patients would only come to hospital once?

Our dataset comprises only hospitalized patients. Blood samples from the same patient were obtained at different days during hospitalization (Npatients=147, Nsamples:1,954). In order to clarify this, we have rephrased the corresponding Result accordingly (**Lines 137-162**). Also, the Method section “Study design and sample collection” gives further details on our patient cohort (**Lines 507-528**). We hope this helps clarify some of the misunderstandings related to the data set.

For any given blood sample (obtained from a hospitalized patient at one particular day) we measured up to eight biomarkers (anti-SARS CoV-2 spike IgG1 antibody titers, white blood cell -WBC- counts, lymphocyte counts, neutrophil counts, eosinophil counts, platelet count, C-reactive protein -CRP-, and blood urea nitrogen -BUN-). Whenever possible, we measured all eight biomarkers in each sample but this was not always possible. The color code in Figure 1A corresponds to the number of biomarkers measured in a particular blood sample of a patient at a particular day. We apologize if this was not clear enough, we have replaced “Clinical features” by “Biomarkers” in figure 1A, next to the corresponding color bar. Also, we have now updated the method section “Sample collection, handling and longitudinally monitored biomarkers” (**Lines 530-539**) and the legend in figure 1: “*(A) Heatmap describing the number of biomarker (y-axis; up to eight different biomarkers, see Methods) measured for any given patient (x-axis; described by an internal identifier) and day (after onset of symptom) over the course of hospitalization (patients=147, blood samples=1,945)*”.

Should the reviewer not find our explanation satisfactory, we will happily work further on it to make it clearer.

- “Current models to predict COVID-19 severity rely on static features obtained upon hospitalised” – a quick literature search seems to bring up a lot of models that have used longitudinal hospitalisation to parameterise their model and predict disease severity see for examples:
  o <https://doi.org/10.1093/cid/ciaa574>
  o DOI: 10.7554/eLife.64827
  o <https://doi.org/10.1186/s13054-020-03255-0>
  o <https://doi.org/10.1371/journal.ppat.1009753>
  o Doi:10.1002/psp4.12574
  And there were more. I think a more comprehensive literature search needs to be done as well as more background given to what is currently being done in this space as there seems to be other relevant works out there. In turn, it would be good for the authors to point out how their work differs to previous studies.

We thank the reviewer for pointing us towards additional papers. We have now added a new section in the introduction where we give an overview of the current work being done around the biological question addressed in this manuscript. We specifically focus on longitudinal studies, which mostly focus on profiling the longitudinal information rather than on predicting disease outcomes. This new section can be found at **lines 96-106**.

- “The trend of higher antibody responses in patients with severe symptoms compared to lower antibody responses in patients with mild-to-moderate symptoms” is an interesting finding. Is there any references or other clinical measurements that support this finding and does it have a biological explanation?

Yes, some other groups also observed a similar trend, not only with anti-Spike IgG but for anti-RBD IgG, anti-NP IgG and even IgM. Regarding biological explanation, the common answer points at the quality of the antibodies produced. Garcia-Belham et al showed that patients with a high anti-RDB IgG neutralizing antibodies potency were more likely to survive. Chakraborty et al observed that severe patients produced more of IgG1 with afucosylated Fc, which are linked to enhanced production of cytokines via Fc effector function. We describe these observations and potential biological explanations and reference these papers in the discussion between line 392 and line 403.

- Figure 2E and then the results in Fig 3 suggest that non-survivors experienced low sustained IgG symptoms (especially earlier), whereas severe disease patients experienced sustained IgG symptoms. Can this be explained? Or a biological explanation hypothesised?

One major observation from our manuscript is that non-survivors display a delayed anti-spike IgG immune response. Zohar et al also observed a similar trend in their patient cohort. This is a key finding and supports the use of therapeutics to enhance antibody response early post symptom onset. A clinical trial where COVID-19 convalescent plasma showed efficacy was in the setting of administration during early post symptom onset (DOI: 10.1056/NEJMoa20337). However, the mechanisms of late antibody response in patients with severe disease is poorly understood. We hypothesized in the discussion (**line 386-390**) that this delay could be due to a lack of IgG class switching as proposed in https://doi.org/10.1016/j.cell.2020.10.052.

- Leading on from the last comment, there is no reference to how the ML techniques utilised in this work have been used elsewhere and how reliable these are as a predictive tool. I think this should be addressed for readers so that they have a point of reference for the computational model presented.

We agree that our rationale for using ML to analyze this dataset and our specific methods were insufficiently detailed in the original manuscript. To address this shortcoming, we have updated our introduction to detail related work in the literature and to specify the methods used (**lines 96-106**) . We have also attached a supplemental methods section containing formal model descriptions and additional citations to the technical aspects of our methods (Supp file 3).

- “Machine Learning models trained on longitudinally monitored immune features outperformed models trained on static features when predicting fatal outcome”. Fatal outcome is just one possible outcome, how can these models be better used to predict optimal therapeutic procotols?

We thank the reviewer for this comment. We propose that machine learning could help early identify patients who are predicted to have severe disease and need ICU care. To inform therapeutic decisions, ML approaches could be integrated into clinical trials to develop a set of biomarkers that predict a therapeutic response. This panel of biomarkers can be monitored during clinical care and when a threshold is achieved alert the clinician, who might then consider a different therapy. In the discussion last paragraph, we are expending on how these models could be better used.

- Is 147 a significant sample size for us to infer results? In turn, could this be a reason that it isn’t possible to distinguish markers for severe-mild patients in most cases?

The reviewer has raised a valid point. The size of the patient cohort can indeed be an issue while looking for patterns distinguishing one group from the others using only admission data. This, however, is not the case for the longitudinal classifier as we have nearly 2,000 data points to train and evaluate our models.

The random forest trained on admission data reports a reasonable AUC at predicting severe patients (AUC=66%) and non-survivors (AUC=63.2%). Predicting patients with mild disease (AUC=46.1%) is an issue as we have only 9 patients in this category. Despite this limitation, we were still able to identify biomarkers already shown in the literature to be relevant (**Lines 303-306**): “*Other features known to be associated to COVID-19 severity and mortality are also predictors of specific categories in our cohort.... For instance, age appears among the top features when predicting mild and severe symptoms, while levels of albumin and lymphocytes predict severe symptoms and mortality*”.

We have acknowledged at the end of the discussion this limitation (**Lines 461-464**): “*An additional limitation of this study is the size of our cohort (Npatients=147). Since the main objective of our study is to evaluate the potential to predict fatal outcomes using only longitudinally monitored biomarkers, we opted for a detailed characterization of the biomarkers trajectories throughout the hospitalization stay (Nsamples= 1,945)*”. We hope the reviewer finds this satisfactory.

- In Fig 6 where “for IgG, we observed a quadratic relationship where both high and low values were associated with a higher propensity of fatal outcomes relative to a middle value” is this statistically significant, as there seems to be a large variation in the Shapley value for the IgG EC50. In turn, what is the “middle value”? Can this be given more specifically?

The interpretation of a Shapely value is the relative amount that a given variable at a given value contributed to a given prediction of the model. This is a local attribution and is a function of the specific input. Hence, there is no unique notion of a ‘middle value’. There are a variety of methods for extracting global attributions from Shapely values but our focus here was to simply demonstrate a trend between the value of immune markers and mortality prediction. We have attached the summary statistics of regressions we performed to the scatterplots we made (Supp File 4). In particular, for the quadratic regression between Shapley value and lab value of EC50, we found a p-value of 9.2*10^(-114) for an F-test and all the parameters individually were significant. This suggests that there is indeed a quadratic relationship between the points we observed.

- Is longitudinal data collection of hospitalised patients a feasible clinical protocol? And is there a way that the authors could determine if there are crucial cut-off/threshold cut-off times for which actual predications can be made for a particular patient. E.g. if a patient is exhibiting the markers of fatal disease after 5 days can we then say with some surety their disease progression and suggest intervention protocols? I think this may be what Figure 5C is showing but could more be expanded on this significance and how it could be used clinically.

With the exception of IgG titers, all other longitudinally followed markers are already part of the routine tests carried out in hospitals. We thus believe it is feasible that longitudinal analysis of clinical data could be automated to feed into a prediction model that feeds back to the clinician in real time.

The AUC reported on figure 5C reflects the confidence on the mortality prediction. Evaluation of our model suggests that predictions are more reliable after the first two weeks post symptom onset. However, as described in the discussion section (**lines 453-469**), the model presented here suffers from certain limitations, such as cohort size and unintended patient selection bias, that would need to be considered in a larger, multicenter study before our model could be used in a clinical setting. Depending on the desired tradeoff between sensitivity and specificity, we could potentially use our model to produce a diagnostic/warning tool that will identify the likely fatal cases within the next few days while having an acceptable false positive/negative rate. This, however, falls outside the scope of our study where the main goal is to show the predictive power of longitudinally monitored biomarkers (specifically immune biomarkers).

Minor

- Is there a motivation for the choice of the different immunological measurements? E.g. why weren’t cytokine measurements taken?

Cytokines degrade rapidly if not kept at 4C after sample collection. The sera we received were kept at room temperature several hours after collection therefore affecting cytokine levels and rendering them unreliable.

- In Fig 1B, could you label the Color bar of the Choropleth map in the figure not just in the caption?

We thank the reviewer for pointing this out, the color bar of the Choropleth map has been labelled.

- I think a legend like that on in Supplementary Figure 3 would be helpful for Figure 3C

As suggested by the reviewer we have updated the legend for Fig 3C accordingly: “*(C) Box-plot at day 18 after onset of symptoms (representative day of the statistical differences observed from days 18 to 24) describing IgG titers (-log_10_ EC_50_) by patient outcome: survival patients requiring oxygen supplementation (nasal cannula: grey, non-rebreather mask: blue; high-flow: salmon; intubation: pink) and non-survival patients (red).*”

- In “…non-survival patients from days 18 to 23 (Fig 3C-D and Supp Fig 3)” it says “Fig 3C-D” but I can’t see a Fig 3D? Is this also the right reference as Fig 3C seems to be distinguishing the patients based on whether they required different oxygen supplies, not on their survival?

The reviewer is absolutely right and we thank him/her for the careful evaluation. We have removed the reference to figure 3D from the text. Both Figure 3C and supplemental Figure 5 (former Supp Fig 3 renumbered) show IgG titers for survivors (further sub-classified according to the type of oxygen type administered) and non-survivors (in red). We have updated both figure legends to clarify this.

- A legend for Fig 4 that is on the figure would be helpful for the reader, so they don’t have to read the caption to determine what the colours mean

As suggested by the reviewer, we have added in the figure a legend describing which line color corresponds to which group of patients.

- In “… status within the next k days using immune biomarkers that would have been available on a given day, as we had these data available (Fig 5B-D).” Should this just be Fig 5C-D as Fig 5B seems to be about week by week ROC on a neural network. If not then it needs to be clearer in the text what Figure 5B is.

Figure 5B describes the weekly ROC curve when predicting mortality within the next five days from the day of evaluation (k=5). We chose this parameter as a representative example and broke down the overall predictive performance by week relative to the day of symptom onset. We noticed that we missed a reference for Fig 5B, while describing the performance of the representative k, which we have now included (**Lines 318-321**). In addition, we have updated the corresponding legend in Fig 5B to further clarify this.

- I’m confused on how the prediction of “death and intubation” is represented in Figure 5, is this in a combined statistic? Or is just one of them represented in Fig 5? If so which one?

We have identified the line in the result section that misguided the reviewer. Figure 5 reports only the performance of predicting mortality. A separate line refers now to the evaluation of intubation prediction (**Lines 321-322**): “*When predicting intubation, the performance of the classifier decreases (AUC= 60.3%; Supp Fig 11C-D)*”

- Is there a label for the Fig 5C color bar?

Following the reviewer’s suggestion, we have added a label for the color bar in Figure 5C

- What do the red diamonds represent in Figure 6E?

The red diamonds highlight samples from patients who died within 5 days of the particular sample. This has been updated in the legend.

- In Fig 4, the mild dynamics become strange between day 11 and 23, is this because the cohort has been reduced to only 1 patient? The reason for the data nonlinearity should be noted somewhere in the text.

Once again, we would like to thank the reviewer for such a careful evaluation. Indeed, the group size of the mild category after day 11 ranges between one to three patients depending on the day (e.g. day 11: 4 patients with available lymphocyte counts, day 14: 1 patient with available lymphocyte counts). We have noted this in the legend of the corresponding figure.

**Reviewer #4:** The authors investigate the use of different biomarkers to model severe disease outcome for COVID-19, including mortality. This study is novel, as it uses longitudinal data of multiple biomarkers to predict mortality and intubation.

The longitudinal approach that the authors take is both interesting and promising. The manuscript is well written and clear for most parts.

I do have some major remarks with this work:

- 1. The dataset used is quite small, which is understandable, as it is harder to collect longitudinal data. However, unless I missed it, I believe there was no power analysis conducted in this work. I understand that this would also be non-trivial, especially for the longitudinal classifier, yet, some measure of uncertainty on the ROC curves could improve this (e.g. by computing the ROC for models from different test/train splits?).

We generated ROC curves after doing 100 cross validation experiments in which we resampled train/test splits each time. We drew an ROC curve for each of those and constructed confidence intervals on a mean ROC curve. Those are indicated on the corresponding figures (Figs 5, S9 and S11) now to demonstrate the confidence in our findings.

- 2. Did the authors consider the effect of the imbalance in data between mortality and survival?

During training, we oversample the minority class by randomly resampling points from each class until we have an even distribution. We do not resample the test set, as this would bias the evaluation of the model. However, the imbalance of the classes in the test set should not affect the ROC curve because ROC curves are invariant to the distribution of true and false labels.

- 3. My main concern with this work is about the longitudinal classifier. I did not find that this model was well explained and I believe that a formal description is necessary. From the current description it was not clear how the trajectory for a patient up until time t, is passed to the model. As all patients have a different trajectory (i.e., they were admitted and left the hospital at different times), I wonder how this is taken into account. It is also not clear how the temporal relationship between the datapoints is modelled. The authors mention the use of a 2-layer neural network, but I wonder whether a LSTM or a temporal convnet was considered, to incorporate the temporal nature of the data.

We agree that our rationale for using ML to analyze this dataset and our specific methods were insufficiently detailed in the original manuscript. To address this shortcoming, we have updated our introduction to detail related work in the literature and to specify the methods used (**lines 96-106**) We have also included a formal longitudinal model description in supplemental methods (Supp file 3) to clarify how we constructed our models. Last we have updated the paragraph about longitudinal immune biomarkers: “*Daily immune biomarkers can predict COVID-19 events on a day-to-day basis”* line 312 and the legend of figure 5 to clarify how patients with different trajectories were integrated in the model.

Also given the size of our data set, we found it difficult to train more advanced models that utilize the entire trajectory to make predictions. We did train an LSTM neural network early on in our experimenting, and we did not get good results. Hence, the final model simply uses the latest available lab values on a given day.

- 4. The authors consider a temporal analysis of the longitudinal classifier, which I think is very useful. However, it would be interesting to include a measure of uncertainty in the predictions, and show how this uncertainty changes as more data becomes available, during a patient’s individual trajectory.

As noted in the prior reviewer comment, our method does not utilize the entire historical trajectory of a patient’s immune markers but rather only considers the last point. Hence, the amount of data that is available does not factor into the model. However, we do quantify the uncertainty in our models; all of our experiments were done in replicate and we plotted our ROC and PRC curves with error bars which show variance across model runs. Those are indicated on the corresponding figures (Figs 5, S9 and S11).

- 5. Adjacent to the remark about the temporal analysis, I was not fully certain whether all data collected up to the point that is to be predicted, is taken into account. Can the authors confirm this and perhaps clarify this in the manuscript? If this is not the case, I worry that the increasing prediction power with respect to mortality (Fig 5B) can be caused by the increasing likelihood of mortality, the longer individuals stay in the hospital.

We experimented with models that take into account the entire trajectory of a patient’s immune markers, such as LSTM recurrent neural networks, as noted in our response to the prior two comments. However, we did not get good results, potentially due to the size of our dataset. It is much more difficult to effectively embed an entire sequence than it is to embed individual points. For this reason, we modeled the problem as one of predicting future mortality from the current time point and were able to get much better results given our available data. One possible interpretation of this observation is that patient outcomes may be roughly Markovian, with much of the information necessary to make the prediction is enclosed within the patient’s current information. Lastly, we want to note that ROC curves are invariant to the distribution of positive and negative samples in a test set; hence, the increasing likelihood of mortality over time cannot explain the increase in predictive performance.

- 6. The authors state “However, there is a need for orthogonal approaches that leverage longitudinal information to inform the early assessment of fatality risk in patients that do not yet show severe signs of disease (35).”, with which I agree. Yet, the models that were fitted on early data (1 week) exhibit poor performance (ROC curves Fig 5 B), so this does not really support this need? Furthermore, the patient cohort considered here exists out of individuals that were already hospitalized. Therefore, I assume that they show severe signs of disease already, so there are no experiments to backup up predicting disease severity in patients with no signs of severe disease?

We thank the reviewer for the thoughtful comments. Early prognosis does not imply that prognosis necessarily needs to be done during week 1 post-symptom onset (PSO) . Nevertheless, while responding to reviewer comments we re-ran our models using days PSO to be consistent with the rest of our analyses and found the AUC for predicting mortality increased to 60.2% when evaluating our model during the first week PSO. The corrected predictive performance is in range with that obtained using only data upon admission (63.2%). In addition, Figures 5C and 5D shows that as k (days into the future) increases, the predictive performance of our model also tends to increase, which supports the suitability of our approach to assist in early prognosis.

With respect to the second half of the comment, our cohort of hospitalized patients with COVID-19 did display a range of disease severity (this was early in the pandemic, when patients with mild disease were hospitalized): 9 patients with mild severity (all survivors), 68 patients with moderate disease (9 of whom did not survive) and 72 patients with severe disease (30 of whom did not survive). Considering that our cohort comprises patients with different degrees of disease severity, we believe our classifier trained on admission values to predict severity of symptoms is valid. It is a good discussion point that a cohort of outpatients could be studied in a similar way to determine if we can predict their outcomes, and this is now added to the discussion (**lines 464-466**): “*And, although our dataset represents the full spectrum of COVID-19 disease, it would be interesting to also consider outpatients to be longitudinally monitored in a similar fashion.*”.

- 7. There was one result I could not wrap my mind around: Fig 5 panel A vs B. In A the red curve corresponds to predicting mortality using static features, while in B the blue curve corresponds to predicting mortality using biomarkers from the first week. While I understand that these models are not exactly the same, yet, I would expect similar performance, as in panel B (1 week) you are using “more” data than in A (just admission data).

The longitudinal model is answering a different question from the baseline classifier. It is not only predicting an outcome, but *when*. It has to decide if an outcome will happen within the next k days. We have clarified the legend of figure 5 panel A and B. Also the baseline classifier has another advantage: it can utilize epidemiological information about the overall likelihood of mortality for patients in the study population as a prior.

- 8. From a technical perspective, on line 470, you mention the number of iterations used. Did you also conduct a convergence analysis to support these values?

The number of iterations described was the maximum number before training would stop. We chose a value large enough that the training would almost always stop on its own before this value would be hit using the default stopping criteria of the optimizer used (I-BFGS or Adam). This point is clarified in the Supp file 3 section 2.2.1.

Minor remarks:

- - One line 213, the authors report a set of biomarkers, please explain based on which criteria this selection was made.

From the patient electronic records, a large set of binary variables (ie: male, sore throat...) and numerical value (age, admission BUN, max eosinophil...) were available. Using logistic regression, we scored those features to predict the outcome of intubation or mortality (see Supp file 3 for more details). The most relevant features (biomarkers) were selected for longitudinal analysis.

- - I found the fact that the methods section was at the end of the paper a bit inconvenient, as I needed to go back and forth while going to the results section. Perhaps some restructuring, or some repetition in the results section could help.

We agree that it’s inconvenient to go back and forth between the results section and the material and method, however we are following the required PLOS Computational Biology format.

**Have the authors made all data and (if applicable) computational code underlying the findings in their manuscript fully available?**
The [PLOS Data policy](https://nam04.safelinks.protection.outlook.com/?url=https%3A%2F%2Fjournals.plos.org%2Fploscompbiol%2Fs%2Fmaterials-and-software-sharing&data=04%7C01%7Colivia.vergnolle%40einsteinmed.org%7Cc9ae931979d843971a3108d97a14f0ca%7C9c01f0fd65e040c089a82dfd51e62025%7C0%7C0%7C637675052638681446%7CUnknown%7CTWFpbGZsb3d8eyJWIjoiMC4wLjAwMDAiLCJQIjoiV2luMzIiLCJBTiI6Ik1haWwiLCJXVCI6Mn0%3D%7C1000&sdata=DBGv3mkSZaUYMfRnw2FOgXFhhlqhaqv%2F4Po7DZDA%2FSk%3D&reserved=0) requires authors to make all data and code underlying the findings described in their manuscript fully available without restriction, with rare exception (please refer to the Data Availability Statement in the manuscript PDF file). The data and code should be provided as part of the manuscript or its supporting information, or deposited to a public repository. For example, in addition to summary statistics, the data points behind means, medians and variance measures should be available. If there are restrictions on publicly sharing data or code —e.g. participant privacy or use of data from a third party—those must be specified.

Reviewer #1: Yes

Reviewer #2: **No:** Data and scripts are not provided.

Reviewer #3: Yes

Reviewer #4: **No:** The source code of the experiments is available on GitHub. There is also a data folder in the GitHub repo, but I did not find a statement whether all data can be found there.

PLOS authors have the option to publish the peer review history of their article ([what does this mean?](https://nam04.safelinks.protection.outlook.com/?url=https%3A%2F%2Fjournals.plos.org%2Fploscompbiol%2Fs%2Feditorial-and-peer-review-process%23loc-peer-review-history&data=04%7C01%7Colivia.vergnolle%40einsteinmed.org%7Cc9ae931979d843971a3108d97a14f0ca%7C9c01f0fd65e040c089a82dfd51e62025%7C0%7C0%7C637675052638681446%7CUnknown%7CTWFpbGZsb3d8eyJWIjoiMC4wLjAwMDAiLCJQIjoiV2luMzIiLCJBTiI6Ik1haWwiLCJXVCI6Mn0%3D%7C1000&sdata=LGue%2FhWW1BF8u3BOsbuqJSv4%2Ba9c0anLDA76poMvgjo%3D&reserved=0)). If published, this will include your full peer review and any attached files.


If you choose “no”, your identity will remain anonymous but your review may still be made public.


**Do you want your identity to be public for this peer review?** For information about this choice, including consent withdrawal, please see our [Privacy Policy](https://nam04.safelinks.protection.outlook.com/?url=https%3A%2F%2Fwww.plos.org%2Fprivacy-policy&data=04%7C01%7Colivia.vergnolle%40einsteinmed.org%7Cc9ae931979d843971a3108d97a14f0ca%7C9c01f0fd65e040c089a82dfd51e62025%7C0%7C0%7C637675052638691398%7CUnknown%7CTWFpbGZsb3d8eyJWIjoiMC4wLjAwMDAiLCJQIjoiV2luMzIiLCJBTiI6Ik1haWwiLCJXVCI6Mn0%3D%7C1000&sdata=oUBKpE49gLvhQ8FZ20Tr7tRg%2B6JtrgEgVemFxC6i0kI%3D&reserved=0).

Reviewer #1: No

Reviewer #2: No

Reviewer #3: No

Reviewer #4: No

Figure Files:

While revising your submission, please upload your figure files to the Preflight Analysis and Conversion Engine (PACE) digital diagnostic tool, [https://pacev2.apexcovantage.com](https://nam04.safelinks.protection.outlook.com/?url=https%3A%2F%2Fpacev2.apexcovantage.com%2F&data=04%7C01%7Colivia.vergnolle%40einsteinmed.org%7Cc9ae931979d843971a3108d97a14f0ca%7C9c01f0fd65e040c089a82dfd51e62025%7C0%7C0%7C637675052638691398%7CUnknown%7CTWFpbGZsb3d8eyJWIjoiMC4wLjAwMDAiLCJQIjoiV2luMzIiLCJBTiI6Ik1haWwiLCJXVCI6Mn0%3D%7C1000&sdata=TNjNOH3bTCy6NMW%2FNvlfB7eYS2Zt736umpXVsrvNzVg%3D&reserved=0). PACE helps ensure that figures meet PLOS requirements. To use PACE, you must first register as a user. Then, login and navigate to the UPLOAD tab, where you will find detailed instructions on how to use the tool. If you encounter any issues or have any questions when using PACE, please email us at [figures@plos.org](mailto:figures@plos.org).

Data Requirements:

Please note that, as a condition of publication, PLOS' data policy requires that you make available all data used to draw the conclusions outlined in your manuscript. Data must be deposited in an appropriate repository, included within the body of the manuscript, or uploaded as supporting information. This includes all numerical values that were used to generate graphs, histograms etc.. For an example in PLOS Biology see here: <http://www.plosbiology.org/article/info%3Adoi%2F10.1371%2Fjournal.pbio.1001908#s5>.

Reproducibility:

To enhance the reproducibility of your results, we recommend that you deposit your laboratory protocols in [protocols.io](http://protocols.io), where a protocol can be assigned its own identifier (DOI) such that it can be cited independently in the future. Additionally, PLOS ONE offers an option to publish peer-reviewed clinical study protocols. Read more information on sharing protocols at[https://plos.org/protocols?utm_medium=editorial-email&utm_source=authorletters&utm_campaign=protocols](https://nam04.safelinks.protection.outlook.com/?url=https%3A%2F%2Fplos.org%2Fprotocols%3Futm_medium%3Deditorial-email%26utm_source%3Dauthorletters%26utm_campaign%3Dprotocols&data=04%7C01%7Colivia.vergnolle%40einsteinmed.org%7Cc9ae931979d843971a3108d97a14f0ca%7C9c01f0fd65e040c089a82dfd51e62025%7C0%7C0%7C637675052638701357%7CUnknown%7CTWFpbGZsb3d8eyJWIjoiMC4wLjAwMDAiLCJQIjoiV2luMzIiLCJBTiI6Ik1haWwiLCJXVCI6Mn0%3D%7C1000&sdata=TbvWJuSIYjT3afIGoOzq0t0Os0shCGPzN1q13N194Ys%3D&reserved=0)
